# Functional identification of two novel carbohydrate-binding modules of glucuronoxylanase CrXyl30 and their contribution to the lignocellulose saccharification

**DOI:** 10.1186/s13068-023-02290-7

**Published:** 2023-03-08

**Authors:** Jiawen Liu, Jingrong Zhu, Qian Xu, Rui Shi, Cong Liu, Di Sun, Weijie Liu

**Affiliations:** Jiangsu Key Laboratory of Phylogenomics & Comparative Genomics, School of Life Science, Jiangsu Normal University, No.101, Shanghai Road, Tongshan New District, Xuzhou, 221116 Jiangsu China

**Keywords:** CBM fusion, Chimeric enzyme, GH30, Xylan, Saccharification

## Abstract

**Background:**

Glycoside hydrolase (GH) family 30 xylanases are a distinct group of xylanases, most of which have a highly specific catalytic activity for glucuronoxylan. Since GH30 xylanases do not normally carry carbohydrate-binding modules (CBMs), our knowledge of the function of their CBMs is lacking.

**Results:**

In this work, the CBM functions of CrXyl30 were investigated. CrXyl30 was a GH30 glucuronoxylanase containing tandem CBM13 (CrCBM13) and CBM2 (CrCBM2) at its C terminus, which was identified in a lignocellulolytic bacterial consortium previously. Both CBMs could bind insoluble and soluble xylan, with CrCBM13 having binding specificity for the xylan with l-arabinosyl substitutions, whereas CrCBM2 targeted l-arabinosyl side chains themselves. Such binding abilities of these two CBMs were completely different from other CBMs in their respective families. Phylogenetic analysis also suggested that both CrCBM13 and CrCBM2 belong to novel branches. Inspection of the simulated structure of CrCBM13 identified a pocket that just accommodates the side chain of 3(2)-alpha-l-arabinofuranosyl-xylotriose, which forms hydrogen bonds with three of the five amino acid residues involved in ligand interaction. The truncation of either CrCBM13 or CrCBM2 did not alter the substrate specificity and optimal reaction conditions of CrXyl30, whereas truncation of CrCBM2 decreased the k_cat_/K_m_ value by 83% (± 0%). Moreover, the absence of CrCBM2 and CrCBM13 resulted in a 5% (± 1%) and a 7% (± 0%) decrease, respectively, in the amount of reducing sugar released by the synergistic hydrolysis of delignified corncob whose hemicellulose is arabinoglucuronoxylan, respectively. In addition, fusion of CrCBM2 with a GH10 xylanase enhanced its catalytic activity against the branched xylan and improved the synergistic hydrolysis efficiency by more than fivefold when delignified corncob was used as substrate. Such a strong stimulation of hydrolysis resulted from the enhancement of hemicellulose hydrolysis on the one hand, and the cellulose hydrolysis is also improved according to the lignocellulose conversion rate measured by HPLC.

**Conclusions:**

This study identifies the functions of two novel CBMs in CrXyl30 and shows the good potential of such CBMs specific for branched ligands in the development of efficient enzyme preparations.

**Supplementary Information:**

The online version contains supplementary material available at 10.1186/s13068-023-02290-7.

## Background

The decomposition of plant cell wall is an integral part to the global carbon cycle, which is mainly completed by lignocellulolytic microorganisms. Due to the complexity of plant cell wall in component and structure, the microorganisms need to use various lignocellulolytic enzymes working in a synergistic manner to decompose it [1]. For example, the hemicellulose contains rich glycosyl side chains resulting in steric hindrance and thus resists to enzymatic hydrolysis, so fully decomposition of hemicellulose requires glycosidases to remove these glycosyl substitution besides the enzymes acting on backbone [2, 3]. Apart from the catalytic domains, non-catalytic carbohydrate-binding modules (CBMs) also contribute significantly to the lignocellulose decomposition. CBMs are commonly connected to catalytic domains via peptide linkers as independent modules in function and structure [4]. The primary function of CBMs is helping catalytic domains bind to their substrates, thereby improving the catalytic efficiency by the targeting effect and proximity effect [5, 6]. In addition, many CBMs can enhance enzyme thermostability and catalysis processivity and even confer substrate specificity for catalytic domains [7–9]. More interestingly, CBMs can also potentiate the enzymatic properties of chimera when artificially fused to other catalytic domains, which is often used for enzyme modification [10]. Therefore, CBM-contained lignocellulosic enzymes are especially concerned.

CBMs have currently been divided into 94 families based on their similarity in amino acid sequence [11]. Such classification aids in identifying new CBMs and revealing their evolutionary relationships but cannot show the functional difference of CBMs. Another classification divides CBMs into three types, where type A CBMs bind to the crystalline surface of insoluble substrates through hydrophobic amino acid residues while type B and C CBMs bind to the interior and termini of soluble polysaccharide chains through their cleft- or dent-like binding sites, respectively [12]. Although the three-type classification shows the binding abilities of CBMs, it does not reflect CBM diversity in the ligand type. To be specific, the polysaccharides in nature vary in structure, and CBMs have different preference for them, which in turn has different effects on enzymatic hydrolysis. For example, certain type B CBMs preferring branched polysaccharides could accordingly enhance the hydrolysis of such substrates, but some other type B CBMs with the specificity for linear polysaccharides could not promote hydrolysis of the polysaccharides with glycosyl substitutions [13]. Detailed identification of the ligand type of CBMs will facilitate our insight into the diversity of CBM functions, which requires more researches.

Xylanase is one of the most common and important hemicellulases. Most xylanases belong to glycoside hydrolase (GH) family 10 and 11, and they can decompose various kinds of xylans like arabinoxylan, glucuronoxylan and linear xylan into xylooligosaccharide and xylose [14]. Some xylanases belong to other GH families, among which the GH30 xylanases generally exhibit a unique substrate specificity for the xylan containing d-glucuronic acid or 4-o-methyl-d-glucuronic acid ((Me)GA) substitutions, namely glucuronoxylan [15]. Such specificity mainly results from the ionic interaction between the side chains of glucuronoxylan and a certain arginine residue of the xylanase [16]. Although the enzyme activities and the structures of certain GH30 xylanases have been studied [17, 18], functions of their CBMs are poorly understood. One important issue to be solved is that GH30 xylanases are commonly specific for glucuronoxylan while it is unclear whether their CBMs have similar ligand specificity. Currently, the CBMs belonging to family 2, 4, 6, 9, 13, 35 and 60 have been found in GH30 xylanases, among which a CBM6 and a CBM35 do act on the same substrate as the catalytic domain [19, 20]. However, the ligand preference and function of many other CBMs have not been studied.

In this study, we identified a GH30 xylanase, CrXyl30, in a lignocellulolytic bacterial consortium, MMBC-1. CrXyl30 contains a GH30 catalytic domain and two CBMs belonging to family 13 and 2, respectively. The full-length and truncated CrXyl30 were heterologously expressed to study the binding abilities of CBMs and their effects on the catalytic performance of CrXyl30. The sequence and structure features of the two CBMs were also investigated. The two CBMs and a GH10 xylanase were finally employed to construct chimeric enzymes to assess the effects of CBM fusion on enzymatic properties and on the saccharification of lignocellulosic biomass.

## Results and discussion

### The binding abilities of recombinant CBMs

**Fig. 1:**
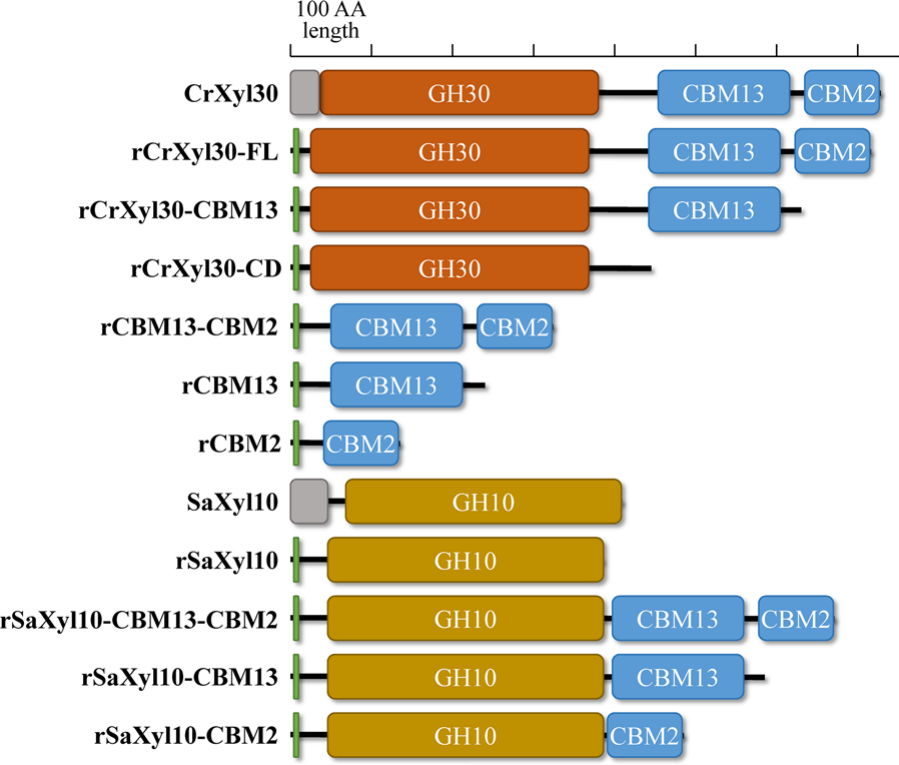
The modular structures of wild-type and recombinant proteins involved in this study. These protein representations are drawn to scale. Gray block: signal peptide; Green block: His-tag; *AA* amino acid

**Fig. 2:**
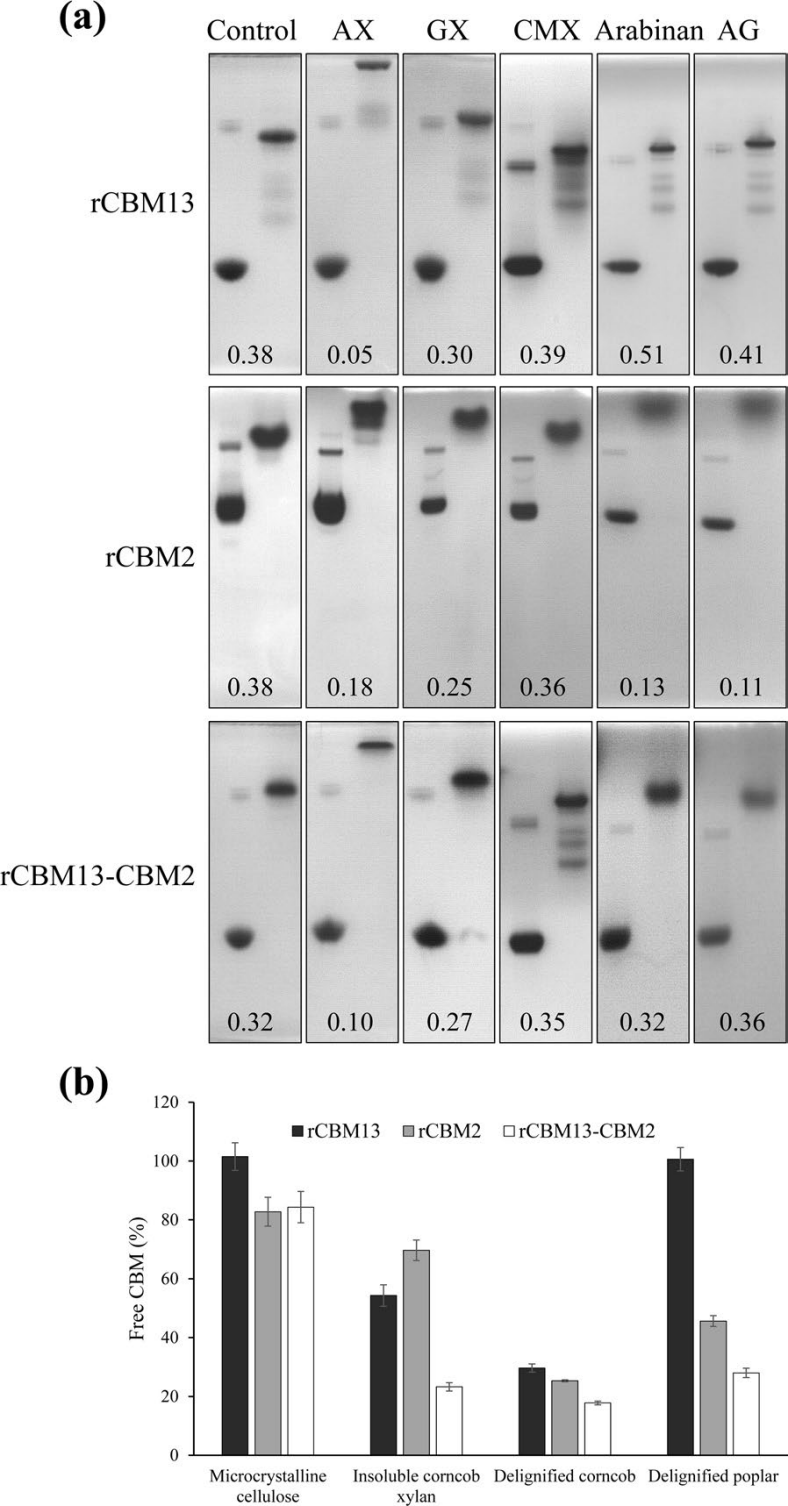
The affinity of recombinant CBMs for **a** soluble polysaccharides and **b** insoluble substrates. Figure 2a shows the non-denaturing PAGE that is carried out in a gel containing arabinoxylan (AX), glucuronoxylan (GX), carboxymethyl xylan (CMX), arabinan or arabinogalactan (AG). The control group contains no polysaccharide. The band of CBM would lag behind that of the control group if the CBM could bind to the corresponding polysaccharide. The numbers indicate the relative mobility rates of CBMs, which are negatively correlated with the affinity of CBMs for polysaccharides. Figure 2b shows the binding abilities of CBMs to microcrystalline cellulose, insoluble corncob xylan, delignified corncob and delignified Carolina poplar. The concentration of the protein solution without any substrate is defined as 100%

The *Crxyl30* gene encoding a xylanase was identified in the metagenome of lignocellulolytic bacterial consortium MMBC-1. It belonged to *Cellulosilyticum ruminicola* according to bioinformatic annotation. The gene was cloned for the heterologous expression of xylanase CrXyl30. CrXyl30 had a distinctive modular structure consisting of a GH30 catalytic domain and two CBMs belonging to family 13 and 2 (termed as CrCBM13 and CrCBM2) (Fig. 1). Enzyme activity analysis of recombinant CrXyl30 revealed its high specificity for glucuronoxylan like most other members of GH30 xylanase (Table 1). To investigate the functions of its two CBMs, a series of truncated CrXyl30 were prepared (Fig. 1). The binding abilities of recombinant CrCBM13 (rCBM13) and CrCBM2 (rCBM2) against arabinoxylan, glucuronoxylan and carboxymethyl xylan were firstly studied through affinity electrophoresis (Fig. 2a). Results showed that the bands of rCBM13 and rCBM2 moved more sluggishly in the gel containing arabinoxylan and glucuronoxylan than in the control group, indicating that both CBMs can bind to these two branched xylans. By comparison, rCBM13 and rCBM2 had no affinity for unbranched carboxymethyl xylan, suggesting that the glycosyl side chains present in xylans are essential to the binding of rCBM13 and rCBM2. Moreover, the dissociation constants (K_d_) of rCBM13 and rCBM2 against arabinoxylan were 2.2 μM and 5.2 μM respectively, which were much smaller than their K_d_ values against glucuronoxylan (28.8 μM and 26.0 μM) (Additional file 1). The relative mobility also showed that rCBM13 and rCBM2, especially the former, had higher affinity for arabinoxylan than glucuronoxylan. To further identify the ligand specificity of CBMs, the affinity of rCBM13 and rCBM2 for arabinan and arabinogalactan which both have L-arabinosyl substitution was studied. Results showed that rCBM2 had strong affinity for arabinan and arabinogalactan, suggesting that this CBM has a binding specificity for arabinose side chain instead of main chain. By comparison, rCBM13 did not bind to arabinan or arabinogalactan, suggesting that this CBM targets the xylan containing arabinosyl substitution. The binding abilities of rCBM13 and rCBM2 against insoluble substrate were then investigated (Fig. 2b). The concentration of rCBM13 in the supernatant decreased significantly after the incubation with insoluble corncob xylan but not with microcrystalline cellulose, indicating that it could bind to insoluble xylan. In comparison, rCBM2 could bind to both microcrystalline cellulose and insoluble xylan.

**Table 1:** The substrate specificity of recombinant CrXyl30

Substrate	rCrXyl30-FL	rCrXyl30-CBM13	rCrXyl30-CD
Glucuronoxylan (U/μmol)	70.7 ± 0.5^a^	55.1 ± 0.7^b^	60.2 ± 0.4^c^
Arabinoxylan	N.D	N.D	N.D
Carboxymethyl xylan	N.D	N.D	N.D
Xylooligosaccharides	N.D	N.D	N.D
Carboxymethyl cellulose	N.D	N.D	N.D
β-1,4-glucan	N.D	N.D	N.D
Arabinan	N.D	N.D	N.D
Arabinogalactan	N.D	N.D	N.D
Galactomannan	N.D	N.D	N.D
Glucomannan	N.D	N.D	N.D
Inulin	N.D	N.D	N.D
Insoluble corncob xylan (mU/μmol)	44.6 ± 2.1^A^	38.2 ± 1.3^B^	39.7 ± 2.4^B^

Different superscripts indicate significant differences (*P* < 0.05)

*FL* full-length; *CD* catalytic domain; *N.D.* Not detected

Both CBMs of CrXyl30 bind to xylan, but the detailed binding characteristics differed. rCBM13 had significant specificity for arabinoxylan, while rCBM2 targeted the L-arabinosyl side chain rather than the main chain of arabinoxylan. Interestingly, the CrXyl30 catalytic domain that acts on glucuronoxylan had a very different binding ability from the two CBMs. In contrast, the catalytic domains and CBMs of other GH30 xylanases appeared to be more functionally coordinated. For examples, the Xyn30D of *Paenibacillus barcinonensis* was specific for glucuronoxylan and its CBM could accordingly bind the (Me)GA side chains of such xylan [20]; the CtXynGH30 of *Clostridium thermocellum* displayed catalytic activity against arabinoxylan and glucuronoxylan, and its CBM6 could also bind both (Me)GA- and arabinose-substituted xylans [21]. The case of CrXyl30 showed that the binding specificity of the CBMs in GH30 xylanase is diverse, which is not necessarily the same as the binding specificity of the catalytic domain. How the CBMs targeting arabinoxylan can enhance the catalytic activity of the GH30 catalytic domain targeting glucuronoxylan needs further study.

### The sequence and structure features of CrCBM13 and CrCBM2

**Fig. 3:**
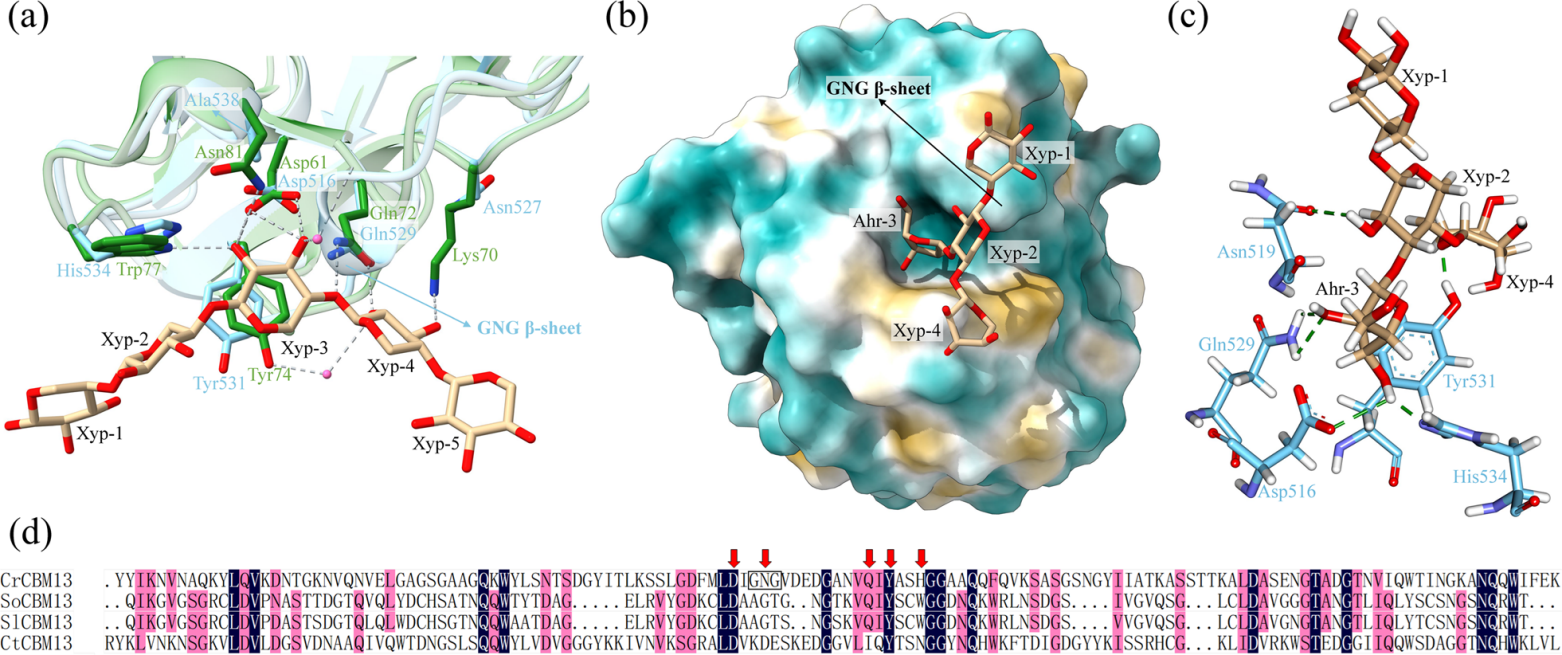
The 3D-structure models of CrCBM13. **a** The structure alignment of SlCBM13-Xylopentaose complex (PDB ID: 1MC9) and CrCBM13. The SlCBM13-Xylopentaose complex and CrCBM13 are colored as dark green and sky blue, respectively. The side chains of the amino acid residues involved in binding xylopentaose in SlCBM13 and of the amino acid residues at the same sequence sites in CrCBM13 are displayed. The pink spheres represent water molecules and the gray dashed lines represent hydrogen bonds. **b** The CrCBM13-Ara*f*-X3 complex based on protein-small molecule docking. Yellow and blue areas represent hydrophobic and hydrophilic surfaces, respectively. **c** The amino acid residues involved in binding Ara*f*-X3 of CrCBM13. The green dashed lines represent hydrogen bonds. **d** Sequence alignment of CBM13. The amino acid residues involved in binding Ara*f*-X3 in CrCBM13 are indicated by red arrows, and the GNG β-sheet is marked with a square. Protein structures of the other three CBMs are accessible at PDB database (1V6V, 1MC9, 3VSZ) [22, 25, 52]

**Fig. 4:**
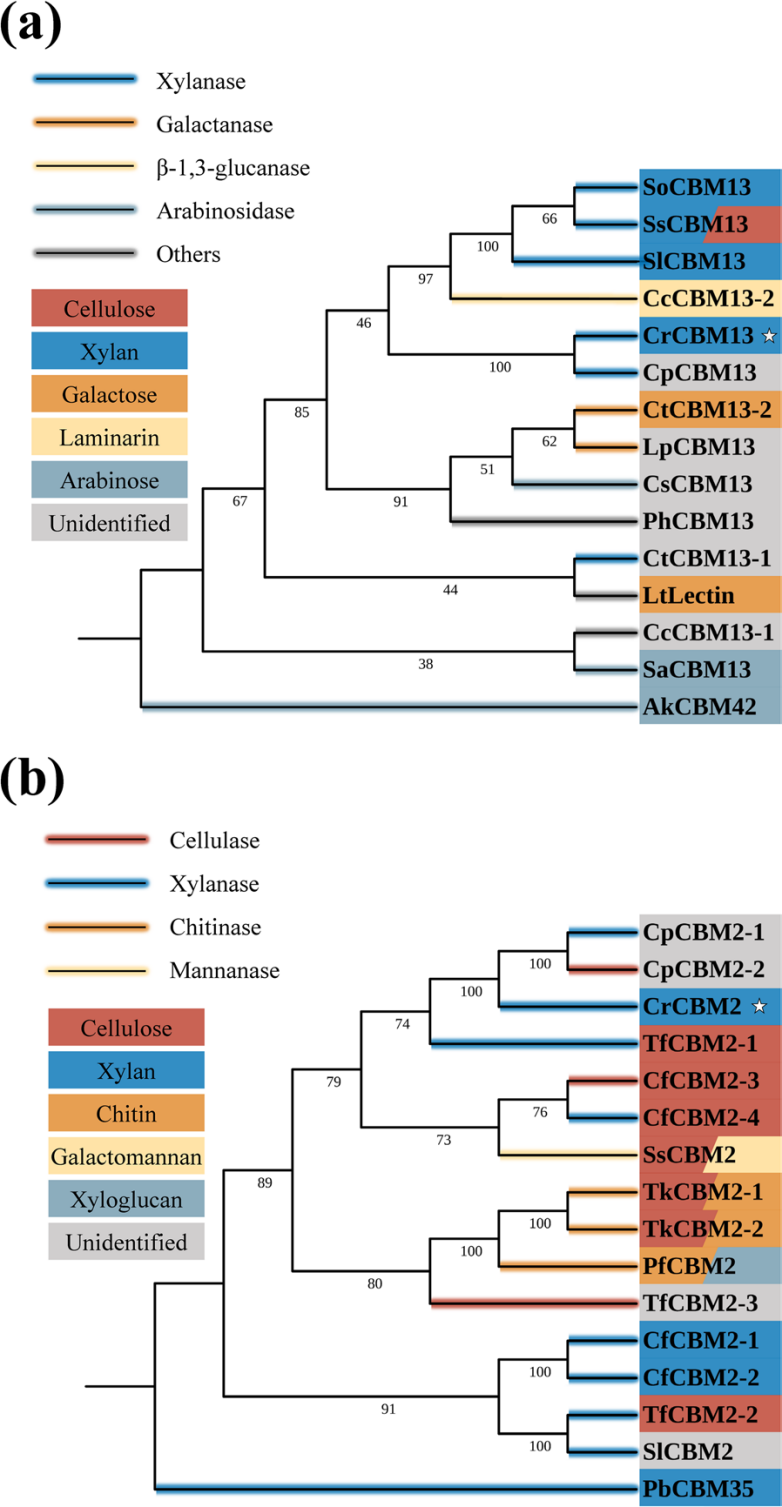
The phylogenetic analysis of **a** CrCBM13 and **b** CrCBM2. The trees mainly include characterized CBMs. The background colors of CBMs indicate their ligand types. The background colors of branches indicate the types of enzymes from which these CBMs are derived. The relevant information was collected from references (see Additional file 7 for details)

The CBMs belonging to family 13 have a broad binding ability against, for examples, linear xylan, galactose, cellulose and arabinose [22–24]. However, no CBM13 specific for arabinoxylan has been found before. To investigate how CrCBM13 binds to arabinoxylan, its 3D-structure was predicted using AlphaFold 2, which was then employed for protein-small molecule docking (Fig. 3 and Additional file 2). Like other members of the CBM13 family, CrCBM13 had a β-trefoil fold, forming three similar subdomains, each of which contains a pocket-like sugar-binding site. However, the binding site of CrCBM13 differs in detail from that of SlCBM13, a CBM13 in the GH10 xylanase of *Streptomyces lividans*, which could bind to linear xylan and xylooligosaccharide [25]. On the one hand, two residues (Lys70 and Asn81) interacting with xylopentaose in SlCBM13 were replaced by Asn527 and Ala538, which could not interact with xylopentaose in CrCBM13. On the other hand, CrCBM13 possessed an additional GNG β-sheet consisting of two glycines and one asparagine, which apparently results in steric resistance to xylopentaose (Fig. 3a). Therefore, CrCBM13 cannot bind to linear xylan. However, the presence of the GNG β-sheet formed a pocket that just accommodated the side chain of 3(2)-alpha-L-arabinofuranosyl-xylotriose (Ara*f*-X3) (Fig. 3b). Further inspection of the CrCBM13-Ara*f*-X3 complex identified five amino acid residues involved in ligand interaction, namely Asp516, Asn519, Gln529, Tyr531 and His534 (Fig. 3c). It is noteworthy that three of the five residues interacted with the side chain of Ara*f*-X3, suggesting that the L-arabinosyl substitution is essential for the interaction with CrCBM13. Compared with other CBMs of family 13, the GNG β-sheet and two out of the five amino acid residues involved in the ligand interaction of CrCBM13 were not conserved (Fig. 3d). The unique binding ability, structural features and phylogenetic position of CrCBM13 suggested that it may represent a new CBM13 subfamily (Fig. 4a).

CBM2 is divided into two subfamilies, CBM2a and CBM2b, based on their sequence and structure features [26, 27]. Compared with CBM2a, CBM2b lacks an eight-residue stretch containing a solvent-exposed tryptophan. CBM2a and CBM2b bind insoluble cellulose and xylan, respectively, and both are type A CBMs. According to protein sequence alignment, CrCBM2 belonged to CBM2a subfamily (Additional file 3), but it targeted both insoluble cellulose and xylan (Fig. 2b). Moreover, it could bind to L-arabinosyl substitution like a type C CBM. CrCBM2 had a similar phylogenetic position to the other two CBM2 of *Clostridium populeti*, and together they formed a previously unstudied branch in the phylogenetic tree (Fig. 4b). In addition, the three CBM2 had some unique sequence features, such as the lack of a conserved glycine and of several contiguous amino acid residues at the C-terminus (Additional file 3), which may explain the unique binding ability of CrCBM2. Therefore, CrCBM2 may be a new member of CBM2a, reflecting the diversity of this CBM subfamily.

### The effects of CBMs on the enzymatic properties of CrXyl30

**Fig. 5:**
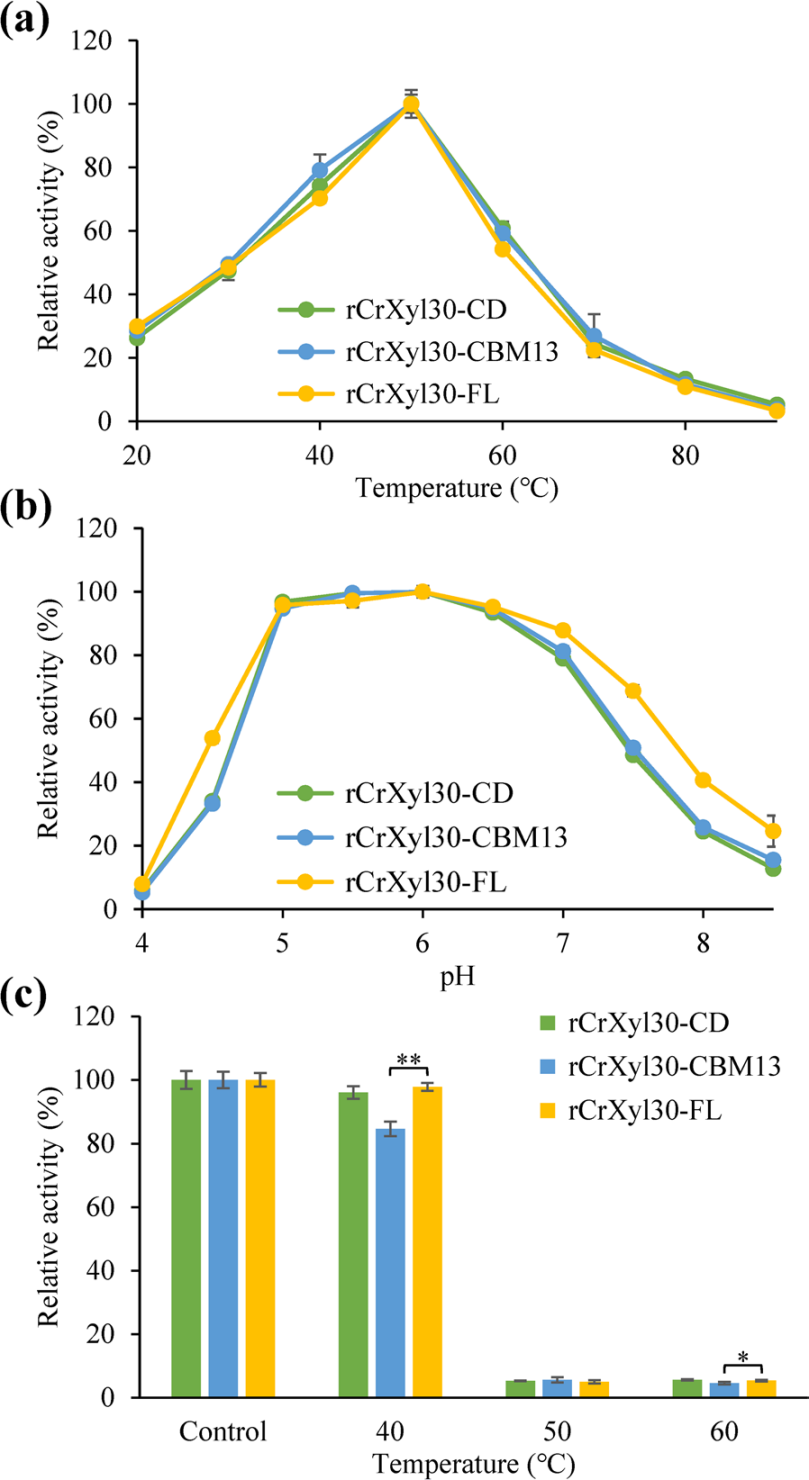
The effects of CBMs on the optimal reaction conditions and the thermostability of CrXyl30. **a** The effects of CBMs on the optimal reaction temperature. **b** The effects of CBMs on the optimal reaction pH value. **c** The effects of CBMs on the thermostability. Significance analysis is performed using *t*-test (*: *P* < 0.05; **: *P* < 0.01). The CBMs of control group were not treated with thermal incubation

The binding abilities between the CBMs and catalytic domain of CrXyl30 are obviously different. To understand the CBM functions, their effects on the optimal catalysis conditions of CrXyl30 were investigated. rCrXyl30-FL (recombinant full-length CrXyl30), rCrXyl30-CBM13 (recombinant CBM2-truncated CrXyl30) and rCrXyl30-CD (recombinant catalytic domain of CrXyl30) all showed their maximal activities at 50 °C (Fig. 5a). Similarly, the three recombinant enzymes had the same optimal catalysis pH value of 6.0 (Fig. 5b). Therefore, CrCBM13 and CrCBM2 did not change the optimal catalysis conditions of CrXyl30. The effects of CBMs on the thermostability of CrXyl30 were then investigated (Fig. 5c). After the incubation at 40 °C for 1 h, the enzyme activity of rCrXyl30-CBM13 decreased by 15.3% (± 2.3%), whereas that of rCrXyl30-FL only slightly decreased (*P* = 0.200) by 2.2% (± 1.2%), indicating that CrCBM2 mildly contributed to the thermostability of CrXyl30. By comparison, the residual activity of rCrXyl30-CD was higher than rCrXyl30-CBM13 after thermal incubation, indicating that CrCBM13 is not helpful to enzyme thermostability.

The effects of CBMs on the kinetic parameters of CrXyl30 were also investigated (Additional file 4). The K_m_ of rCrXyl30-FL, rCrXyl30-CBM13 and rCrXyl30-CD against glucuronoxylan were 0.7 ± 0.0 g/L, 2.0 ± 0.1 g/L and 1.6 ± 0.1 g/L, respectively, indicating that CrCBM2 promotes the binding of CrXyl30 to such substrate but CrCBM13 cannot. Moreover, the k_cat_ of rCrXyl30-FL (11.8 ± 0.4 s^−1^) was significantly higher than that of rCrXyl30-CBM13 (5.5 ± 0.2 s^−1^), indicating that CrCBM2 improves catalytic efficiency as well. However, rCrXyl30-CD also had a higher k_cat_ value (6.4 ± 0.2 s^−1^) than rCrXyl30-CBM13, indicating that CrCBM13 does not contribute to catalytic efficiency. Therefore, although both CBMs had modest binding ability to glucuronoxylan, they exerted distinct effects on enzymatic properties, where CrCBM2 helped CrXyl30 in binding and hydrolyzing glucuronoxylan while CrCBM13 played an unclear role. The effect of CBM on the hydrolysis of soluble polysaccharides is variable. For examples, the CBM35 of Xyn30D did not contribute to the catalytic properties of catalytic domain, while the CBM6 of CtXynGH30 enhanced the enzymatic hydrolysis of both arabinoxylan and glucuronoxylan [20, 28]. The function of CBMs is influenced by many factors, such as the type and concentration of substrate [29]. Therefore, in these studies [20, 28], CBMs have shown no effect on catalysis, probably because the experimental conditions used were not conducive to their function. The biological roles of these “ineffective” CBMs will need to be elucidated by further research using genetic or other methods.

### The effects of CBMs on the synergistic hydrolysis of lignocellulosic biomass

**Fig. 6:**
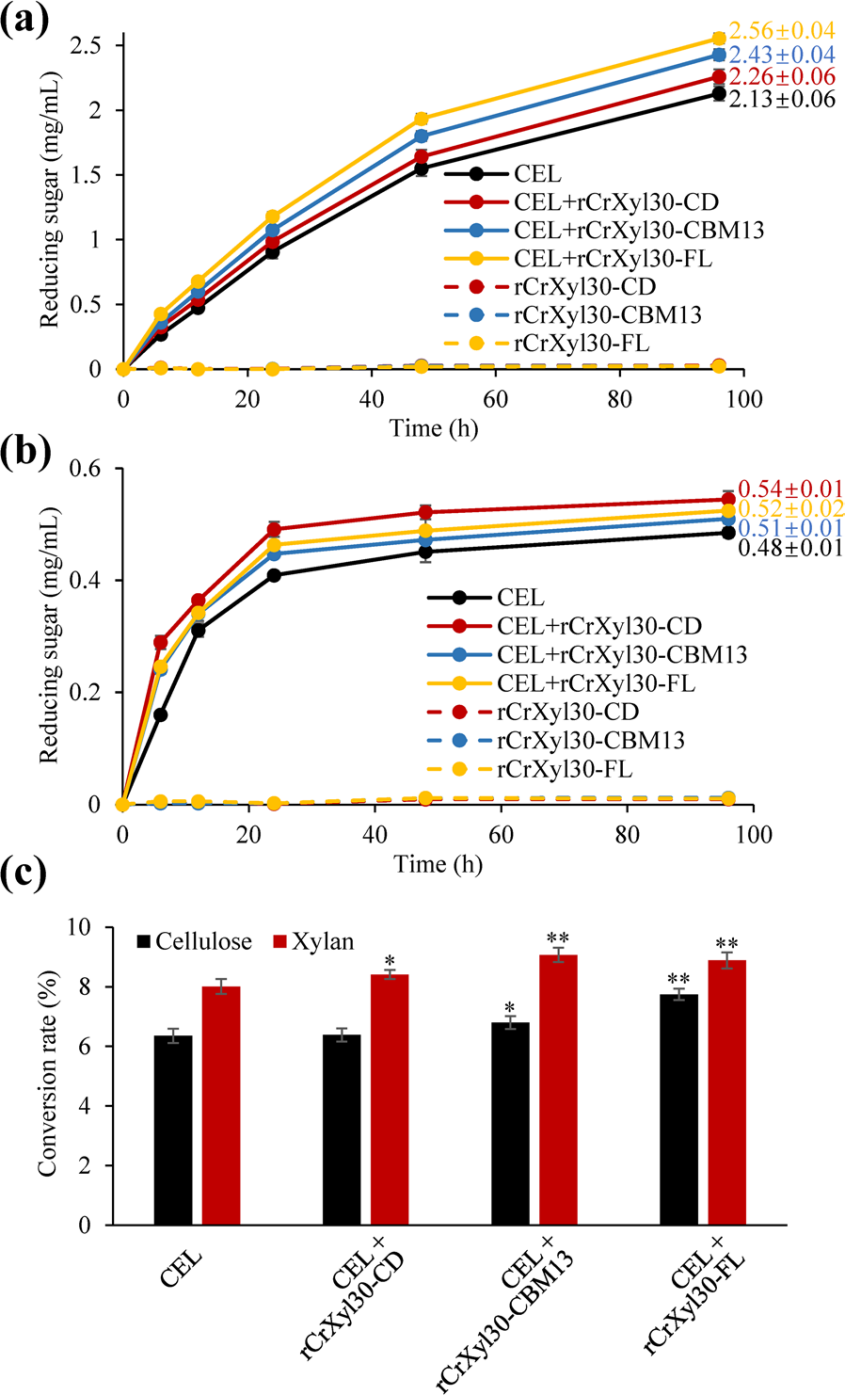
The effects of CBMs on the synergistic hydrolysis by commercial cellulase and recombinant CrXyl30. **a** The synergistic hydrolysis of delignified corncob by CEL and rCrXyl30-FL or its CBM-truncated versions. **b** The synergistic hydrolysis of delignified Carolina poplar by CEL and rCrXyl30-FL or its CBM-truncated versions. **c** The cellulose and xylan conversion rates of delignified corncob at the 96th hour of hydrolysis. The numbers in Fig. 6a, b indicate the reducing sugar concentration (mg/mL) at the 96th hour. In Fig. 6c, the significance analysis of difference between CEL and other groups is performed using *t*-test (*: *P* < 0.05; **: *P* < 0.01)

In natural environment, xylanases hydrolyze lignocellulose together with other carbohydrate-active enzymes in a synergistic way instead of working alone. To investigate the role of CBMs in synergistic hydrolysis, commercial cellulase (CEL), which is derived from the fermentation broth of *Trichoderma reesei*, and full-length or truncated CrXyl30 were employed for the saccharification of delignified corncob and Carolina poplar. When corncob was used as the substrate of CEL, the reducing sugar concentration at the 96th hour was improved from 2.13 ± 0.06 mg/mL to 2.26 ± 0.06 mg/mL by the addition of rCrXyl30-CD (Fig. 6a). By comparison, the reducing sugar concentration increased to 2.43 ± 0.04 mg/mL and 2.56 ± 0.04 mg/mL, respectively, at the 96th hour with the addition of equimolar rCrXyl30-CBM13 and rCrXyl30-FL, indicating that both of CrCBM13 and CrCBM2 could enhance the synergistic hydrolysis of corncob. When Carolina poplar was used, the two CBMs showed different influence on the hydrolysis. Specifically, the reducing sugar concentration was improved from 0.48 ± 0.01 mg/mL to 0.54 ± 0.01 mg/mL at the 96th hour by the addition of rCrXyl30-CD (Fig. 6b). Such enhancement in hydrolysis by rCrXyl30-CD was stronger than that by rCrXyl30-CBM13 (0.51 ± 0.01 mg/mL) and rCrXyl30-FL (0.52 ± 0.02 mg/mL), indicating that the two CBMs cannot stimulate the hydrolysis of Carolina poplar. Correspondingly, the adsorption experiment showed that the two CBMs had stronger affinity for corncob than for Carolina poplar (Fig. 2b), although the trace amounts of residual lignin present in lignocellulosic biomass might affect the adsorption of CBMs. We further studied which of corncob polysaccharide components had their hydrolysis enhanced by xylanase. Results showed that the addition of rCrXyl30-CD only improved the xylan conversion rate, while that of rCrXyl30-CBM13 or rCrXyl30-FL enhanced the conversion rates of both cellulose and xylan (Fig. 6c).

The hemicellulose of corncob is mainly arabinoglucuronoxylan which contains both arabinose and (Me)GA substitutions, while the hemicellulose of Carolina poplar is mainly glucuronoxylan that contains only branched (Me)GA residues [30]. The strong affinity of rCBM13 and rCBM2 for corncob could result from their binding specificity for the arabinoglucuronoxylan with arabinosyl substitutions. As mentioned earlier, the catalytic domain of CrXyl30 had a different binding ability from the two CBMs. Such divergence may be related to the arabinoglucuronoxylan decomposition. Specifically, Crxyl30 belongs to *Cellulosilyticum ruminicola*, which is a rumen bacterium with lignocellulolytic ability, and arabinoglucuronoxylan widely exists in many herbaceous plants that would be degraded in herbivore intestine by lignocellulolytic microorganisms [31]. Some kinds of arabinoglucuronoxylan contain abundant arabinose side chains but is only slightly decorated with (Me)GA, namely glucuronoarabinoxylan. In these cases, an CBM targeting branched L-arabinofuranosyl residues is more conducive to the binding of enzyme to substrate and then strengthen the hydrolysis of (Me)GA-substituted regions near the binding sites by linked catalytic domain. Unlike other reported GH30 xylanases whose CBMs showed the similar binding ability with catalytic domains [20, 21], CrXyl30 may represented another case where the CBMs and catalytic domain bind to different structural sites of the same substrate to enhance the enzymatic hydrolysis. The Xyl10A from *Talaromyces cellulolyticus* had a similar mechanism, where its CBM1 enhanced the xylan hydrolysis by binding to cellulose [32]. Moreover, the C-terminal CBM10 of *Sd*GH5_8-CBM10 × 3 (an endo-β-mannanase) could anchor the enzyme to its substrates or spatially adjacent polysaccharides such as crystalline cellulose, thus ensuring the heterogenous catalysis on insoluble substrates [33]. These cases suggest that the employ of a CBM with divergent binding ability from catalytic domain is a common strategy to promote the synergistic hydrolysis of lignocellulose by microorganisms.

### The effects of CBMs on the enzymatic properties of chimeric xylanase

**Fig. 7:**
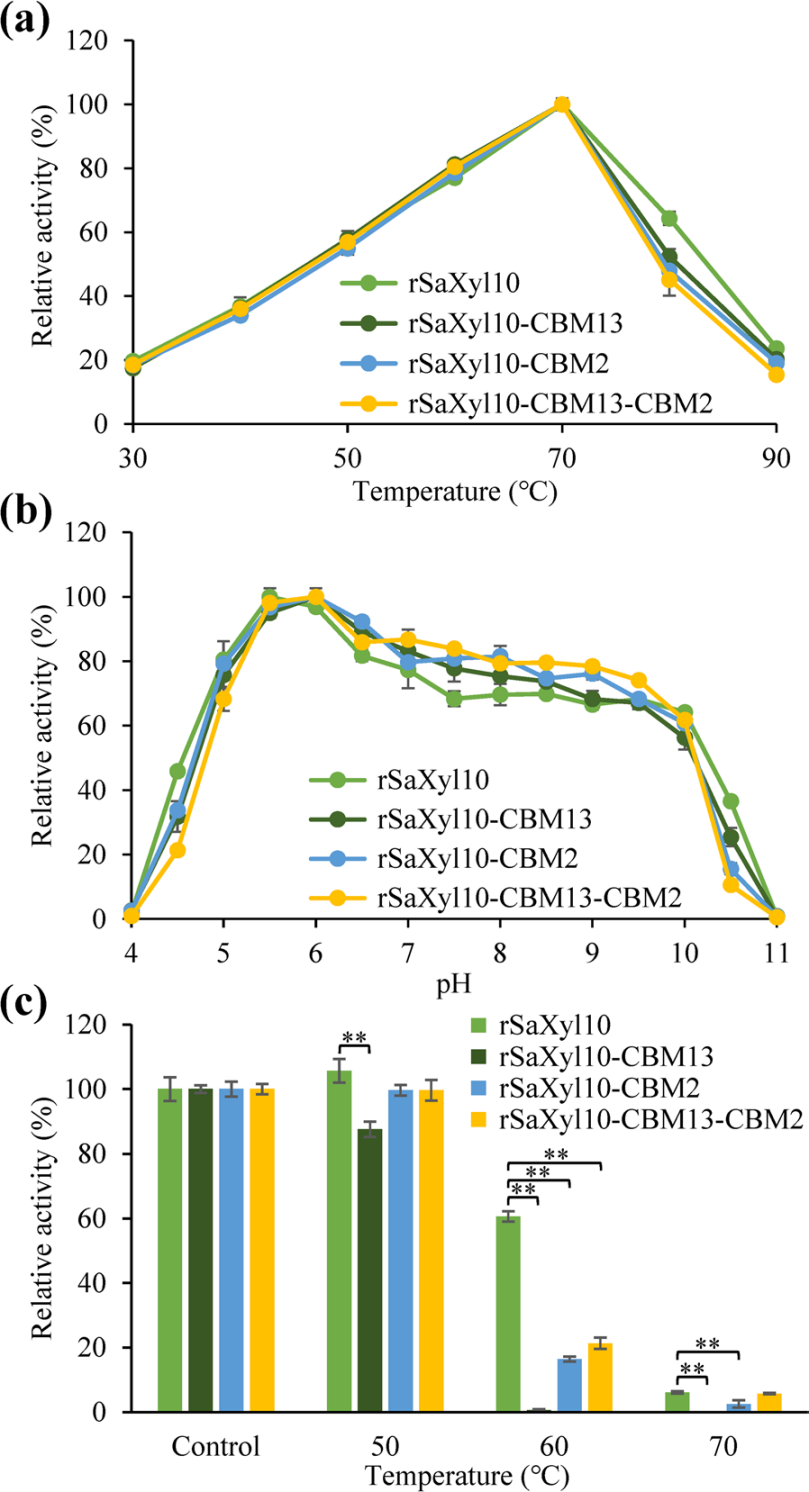
The effects of CBMs on the optimal reaction conditions and the thermostability of SaXyl10. **a** The effects of CBMs on the optimal reaction temperature. **b** The effects of CBMs on the optimal reaction pH value. **c** The effects of CBMs on the thermostability. Significance analysis is performed using *t*-test (*: *P* < 0.05; **: *P* < 0.01). The CBMs of control group were not treated with thermal incubation

In order to deeply digest their effects on the catalytic domain of xylanase, CrCBM13 and CrCBM2 were fused with Saxyl10, a GH10 xylanase with a broad substrate specificity, at its C-terminal to construct three chimeric enzymes (rSaxyl10-CBM13, rSaxyl10-CBM2 and rSaxyl10-CBM13-CBM2) (Fig. 1). Non-fused recombinant Saxyl10 (rSaxyl10) showed its maximal activity at 70 °C and pH5.5 (Fig. 7a, b). The fusion of CBMs did not change the optimal reaction temperature while slightly raised the optimal reaction pH value to 6.0. The CBMs, however, obviously impaired the thermostability of Saxyl10. For example, rSaxyl10 retained 60.7% (± 1.6%) of its initial enzyme activity after the incubation at 60 °C for 1 h, while rSaxyl10-CBM13, rSaxyl10-CBM2 and rSaxyl10-CBM13-CBM2 only retained 0.7% (± 0.2%), 16.5% (± 0.7%) and 21.3% (± 1.8%) of its initial enzyme activity after the same treatment, respectively (Fig. 7c).

The effects of CBMs on the kinetic parameters of Saxyl10 were also investigated (Table 2). None of those chimeric enzymes had statistically smaller K_m_ than rSaxyl10, indicating that neither CrCBM13 nor CrCBM2 improve the affinity of Saxyl10 for glucuronoxylan or arabinoxylan. rSaXyl10-CBM2 had a higher k_cat_ than rSaxyl10, indicating that a single CrCBM2 improves the catalytic efficiency of Saxyl10 against glucuronoxylan and arabinoxylan. In addition, none of these CBMs enhanced the affinity or catalytic efficiency of Saxyl10 against unbranched carboxymethyl xylan. The enhancement of the catalytic efficiency but not the binding ability of Saxyl10 by CrCBM2 was unexpected, and the mechanism cannot be explained on the basis of the current data alone. Despite all this, CrCBM2 only contributed to the hydrolysis of branched xylan, in accordance with its ligand specificity. The invalidity of CrCBM13 may resulted from the use of an inappropriate linker.

**Table 2:**
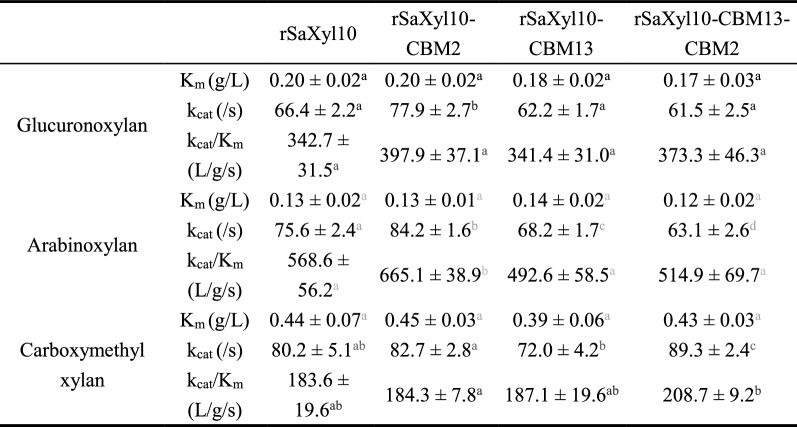
The kinetic parameters of rSaXyl10 and its CBM-fusion versions against xylans

Superscript in the same color shows the results of significant difference analysis, where different lowercase letters indicate significant difference (*P* < 0.05)

### The effects of CBMs on the synergistic hydrolysis using chimeric xylanases

**Fig. 8:**
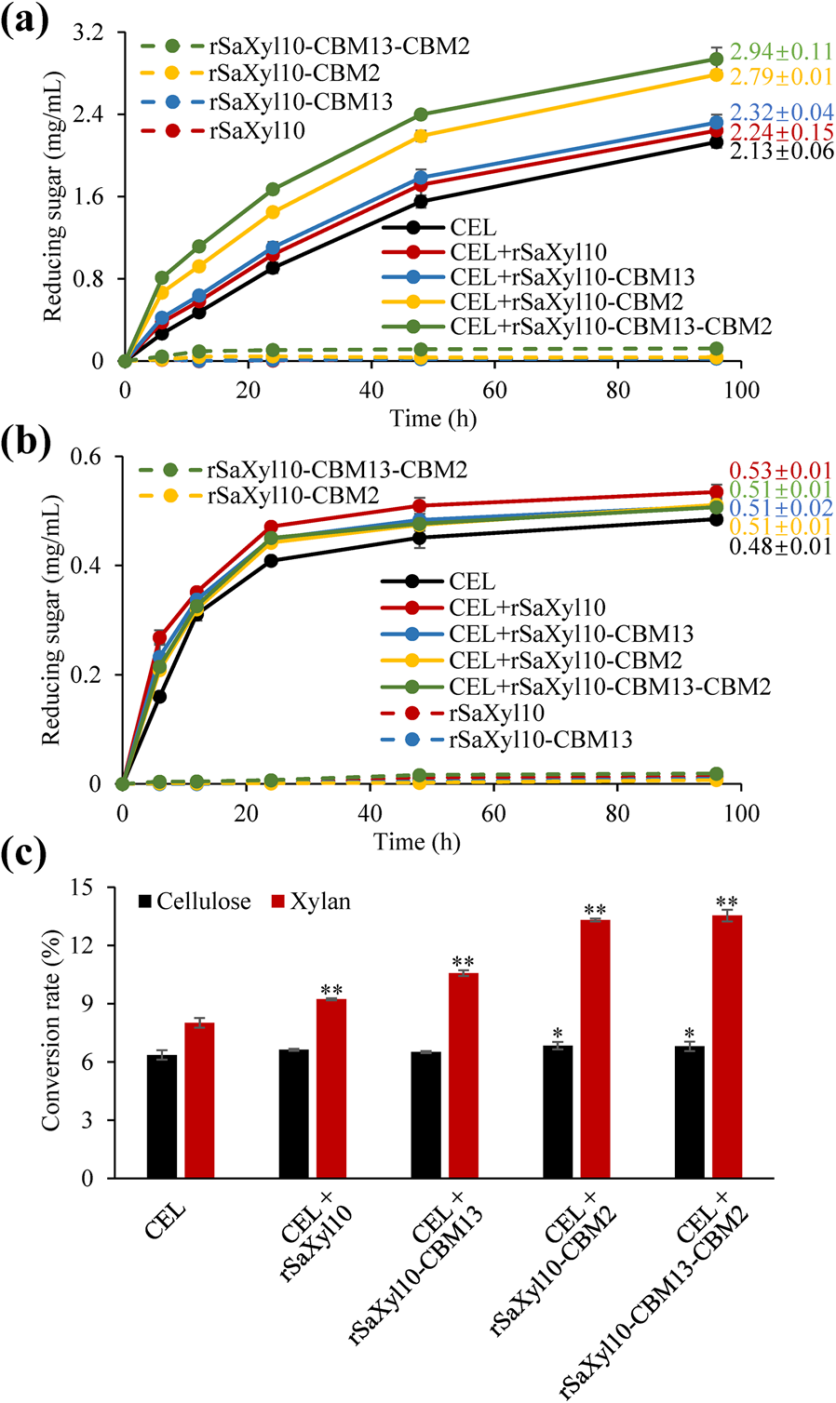
The effects of CBMs on the synergistic hydrolysis by commercial cellulase and recombinant SaXyl10. **a** The synergistic hydrolysis of delignified corncob by CEL and rSaXyl10 or its CBM-fusion versions. **b** The synergistic hydrolysis of delignified Carolina poplar by CEL and rSaXyl10 or its CBM-fusion versions. **c** The cellulose and xylan conversion rates of delignified corncob at the 96th hour of hydrolysis. The numbers in Fig. 8a, b indicate the reducing sugar concentration (mg/mL) at the 96th hour. In Fig. 8c, the significance analysis of difference between CEL and other groups is performed using *t*-test (*: *P* < 0.05; **: *P* < 0.01)

The effects of CrCBM13 and CrCBM2 on the synergistic hydrolysis of lignocellulosic biomass by commercial cellulase (CEL) and rSaxyl10 or chimeric enzymes were also investigated. When delignified corncob was used as the substrate, the reducing sugar concentration slightly increased (*P* = 0.297) by 6% (± 1%) from 2.13 ± 0.06 mg/mL to 2.24 ± 0.15 mg/mL at the 96th hour with the addition of rSaxyl10 (Fig. 8a). The addition of rSaxyl10-CBM13 did not result in statistically higher reducing sugar concentration compared with rSaxyl10, indicating CrCBM13 could hardly contribute to the synergistic hydrolysis. However, the addition of rSaxyl10-CBM2 resulted in a 31% (± 2%) improvement in reducing sugar concentration from 2.13 ± 0.06 mg/mL to 2.79 ± 0.01 mg/mL at the 96th hour. Moreover, the supplement of rSaxyl10-CBM13-CBM2 also led to a huge improvement (38 ± 2%) in reducing sugar concentration, showing the important contribution of CrCBM2 to synergistic hydrolysis. When acting on Carolina poplar, the fusion of the CBMs to Saxyl10 could not further boost the synergistic hydrolysis, which may result from the weak binding abilities of the CBMs to such substrate (Fig. 8b). In addition, both rSaxyl10 and rSaxyl10-CBM13 only enhanced the enzymatic degradation of corncob xylan, while rSaxyl10-CBM2 and rSaxyl10-CBM13-CBM2 additionally stimulated the conversion of corncob cellulose (Fig. 8c).

The contribution of CrCBM2 in chimeric xylanases to the synergistic hydrolysis of corncob is impressive. Such effect results from, on the one hand, the enhancement of hemicellulose hydrolysis. On the other hand, the cellulose hydrolysis is also improved through the proximity and synergistic effects. Improving the hydrolysis efficiency of lignocellulosic biomass by CBM is common. For examples, the additional employ of full-length and CBM-truncated acetyl xylan esterases respectively led to a 17.5% and an 8.0% increase in the xylan conversion rate of NaClO_2_-treated wheat straw compared with the employ of xylanase alone [34]; When sulfuric-acid-pretreated corn bran was used as substrate, the additional use of a full-length xyloglucanase led to an 136.2% increase in reducing sugar yield compared with using cellulases alone, while the addition of CBM-truncated xyloglucanase only increased reducing sugar yield by 48.9% [35]. By comparison, fusion of CrCBM2 enhanced the promotion effect of Saxyl10 on synergistic hydrolysis by more than fivefold (from 6 to 31%), indicating a stronger contribution of the non-catalytic CrCBM2 even than that of the GH10 catalytic domain. Side chain is the main factor that hemicellulose resists to enzymatic degradation. The striking facilitation effect of CrCBM2 may be attributed to its binding to glycosyl substitution sites and then specifically enhancing the hydrolysis efficiency of branch structure, namely accelerating the rate-limiting step [36]. A similar example of fructanase has been reported previously: fusing *Bs*CBM66, a type C CBM targeting the terminal fructoside, to a β-fructosidase resulted in a 120-fold increase in its enzymatic activity against levan, a branched fructan, but led to a negligible influence on that against inulin, a linear fructan [37]. Therefore, present study confirmed the potentiation of xylan hydrolysis by such CBMs targeting branched polysaccharides and suggested their good potential for the development of efficient enzyme preparation for industrial applications. CrCBM13 binds more strongly to arabinoxylan than CrCBM2 (Fig. 2a and Additional file 1), but the synergism experiments show that the contribution of CrCBM2 to arabinoxylan degradation is much higher than that of CBM13 in the chimeric enzyme. The function of CBM is influenced by many factors, such as the orientation between CBM and catalytic domain or the type of linker peptide. The current structure of the chimeric protein may not be conducive to the function of CrCBM13. Further efforts are needed to address this issue.

## Conclusions

This study shows that the CBM of GH30 xylanase is diverse and it does not necessarily target the same substrate as the catalytic domain. CrXyl30 contains two novel CBMs specific for arabinoxylan, which can, however, can significantly promote the corncob hydrolysis by the catalytic domain with a glucuronoxylanase activity. Moreover, the CrCBM2 also has a striking facilitative effect on the catalysis of chimeric GH10 xylanase, suggesting that the fusion of such kind of CBM targeting branched ligands is a promising way to construct efficient enzyme preparation.

## Methods

### Strains and substrates

The lignocellulolytic bacterial consortium (MMBC-1) and *Salipaludibacillus agaradhaerens* C9 (identified as *Bacillus agaradhaerens* previously) were isolated from soil samples and then preserved in our lab [38–41].

Arabinoxylan (9040-27-1), glucuronoxylan (9014-63-5) and xylooligosaccharides (the mixture of xylobiose, xylotriose, xylotetraose, xylopentaose and xylohexaose) were purchased from Megazyme (Ireland). Arabinan (11078–27-6) and arabinogalactan (9036-66-2) were purchased from Psaitong (China) and Macklin Biochemical (Shanghai) Co., Ltd, respectively. Insoluble corncob xylan (9014-63-5) was purchased from Yuanju Biotech (Shanghai) Co., Ltd. and used for the preparation of unbranched carboxymethyl xylan basing on an established method [42, 43]. In brief, 15 g of corncob xylan were dissolved in 900 mL of NaOH solution (0.8 M) and incubated at 60 °C for 3 h. The supernatant was collected by centrifugation and then neutralized with hydrochloric acid. After standing for 2 h, the precipitate was collected by centrifugation and then added into 900 mL of NaOH (0.2 M). The insoluble xylan was unbranched, which was collected and washed to neutral pH. After drying, 2.5 g of unbranched xylan were dispersed in 75 mL of isopropanol and stirred at room temperature for 1 h after the addition of 7.5 mL of NaOH solution (0.5 M). After that, 4.4 g of sodium monochloroacetate were added, followed by stirring at 60 °C for 2 h. The product was redissolved in 50 mL of deionized water and neutralized with acetic acid. After dialysis against water, the carboxymethyl xylan powders were prepared by lyophilization. The other soluble polysaccharides were purchased from Macklin Biochemical (Shanghai) Co., Ltd. The corncob was collected from farmland in Huainan city (China), and Carolina poplar (*Populus* x *canadensis* Moench) was purchased from a logging camp in Beijing, China. To prepare the delignified biomass, corncob and Carolina poplar were both ground firstly. Afterwards, 10 g of powder were dispersed in 100 mL of solution containing 5% NaClO_2_ and 1% acetic acid, followed by incubation at room temperature in dark for 24 h. Such treatment was repeated four more times to fully remove the lignin of corncob and Carolina poplar. Finally, the powders were washed to neutral pH and then dried at 60 °C. The composition of delignified lignocellulosic biomass was measured according to the method offered by National Renewable Energy Laboratory [44] and is shown in Additional file 5.

### The construction of recombinant plasmids

The genes of CrXyl30 and SaXyl10 (accession number: OP172628 and OP172629) were cloned using KOD DNA polymerases (TOYOBO, Japan) from the metagenome DNA of MMBC-1 and genome DNA of *S. agaradhaerens* C9, respectively. The DNA fragments were digested with restriction endonucleases (Additional file 6) and then inserted into the plasmid pET28a ( +) treated with the same digestion. Subsequently, the recombinant plasmid was transformed into *E. coli* DH5α, followed by the identification via PCR and sequencing. The validated recombinant plasmids were extracted and then transformed into *E. coli* BL21(DE3) for the expression of recombinant proteins.

Partial CrXyl30 genes were cloned using corresponding primers to construct truncated enzymes (Additional file 6). For the construction of CBM-fusion enzymes, the genes of Saxyl10 and CBMs were cloned with special primers, resulting in an overlap about 40-bp long between the two DNA fragments (Additional file 6). Afterwards, the Saxyl10 and CBM genes were fused by PCR according to a previous method [45]. Both truncated and chimeric genes were then employed to construct recombinant plasmids basing on the method mentioned earlier.

### The heterologous expression of recombinant proteins

The *E. coli* BL21(DE3) containing recombinant plasmid was cultured in 5 mL of LB-kanamycin medium at 37 °C overnight. The seed culture was then transferred into an Erlenmeyer flask containing 200 mL of LB-kanamycin medium, followed by the incubation in a shaker at 37 °C with a rotational speed of 200 rpm. Isopropy-β-d-thiogalactoside was added into the medium to a final concentration of 0.6 mM when the absorbance value at 600 nm of the medium reached 1.0. Subsequently, the culture temperature was adjusted to 16 °C and cells were harvested after 20 h through centrifugation.

The cells harvested in the previous step were resuspended in 20 mL of binding buffer (20 mM of Tris–HCl, 500 mM of NaCl, pH8.0), followed by cell disruption through sonication. Afterwards, the supernatant was collected via centrifugation and then loaded into a Ni–NTA column. The undesired and recombinant proteins were eluted with washing buffer (20 mM of Tris–HCl, 500 mM of NaCl, 20 mM of imidazole, pH8.0) and elution buffer (20 mM of Tris–HCl, 500 mM of NaCl, 250 mM of imidazole, pH8.0), respectively. The eluent containing recombinant proteins was collected and treated with dialysis against water to remove small molecular weight impurity. The recombinant proteins were finally prepared through lyophilization.

### The analysis of CBM affinity

The binding abilities of CBMs for soluble polysaccharides were investigated through affinity electrophoresis according to an established method [46]. In brief, five micrograms of the recombinant CBMs as well as bovine albumin (BSA) were respectively mixed with loading buffer and then used for non-denaturing polyacrylamide gel electrophoresis (PAGE) in a 10% gel. Soluble polysaccharides (arabinoxylan, glucuronoxylan, carboxymethyl xylan, arabinan or arabinogalactan) were added to a final concentration of 0.1% (w/v) when making separating gels. The electrophoresis was carried out in ice bath for 2 h with a voltage of 150 V, followed by Coomassie blue staining. The relative mobility (r) of CBM (distance migrated by CBM band divided by the distance migrated by dye) was calculated to show the CBM affinity for soluble polysaccharides. To determine the K_d_ of CrCBM13 and CrCBM2, the affinity electrophoresis was carried out using the gels containing arabinoxylan or glucuronoxylan with a gradient concentration (0/0.1/0.2/0.4/0.8 g/L for arabinoxylan and 0/0.75/1.25/2.5/5 g/L for glucuronoxylan). The plots of 1/r versus ligand concentration were then used to determine of K_d_ by regression analysis (Additional file 1), and the unit of K_d_ was finally converted from g/L to μM according to the average molecular weights of arabinoxylan and glucuronoxylan.

To investigate the affinity of CBMs for insoluble substrates, the microcrystalline cellulose, corncob xylan, delignified corncob or Carolina poplar were ground to the powders with particle sizes below 75# mesh. The CBMs and substrates were then dispersed in 1 mL of Na_2_HPO_4_-NaH_2_PO_4_ buffer (100 mM, pH 7.0) to a final concentration of 0.5 mg/mL and 10 mg/mL respectively in a 2 mL low-binding tube (Eppendorf, Germany). Subsequently, the tube was incubated in a shaker at 37 °C with a rotational speed of 200 rpm for 1 h. The supernatant was then collected through centrifugation, followed by the determination of protein concentration using Bradford assay. The concentration of the protein solution without any substrate is defined as 100%.

### Enzyme assays

To investigate the specific activities of the enzymes, the recombinant xylanases and soluble polysaccharides were dissolved in the disodium hydrogen phosphate—citric acid buffer (pH 6.0, 200 mM). After that, 50 μL of recombinant xylanase solution (5 μM) was mixed with 100 μL of polysaccharide solution (0.5%) and incubated at 50 °C for 10 min. Afterwards, 200 μL of dinitrosalicylic acid (DNS) reagent was added, and the mixture was incubated in a boiling water bath for 5 min [47]. Subsequently, 1 mL of deionized water was added to dilute the solution. The supernatant was collected after centrifugation, and its absorbance at 520 nm was measured to calculate the reducing sugar concentration and the enzyme activity according to the calibration curve using corresponding monosaccharides as standard. The amount of enzyme required to produce 1 μmol of product per minute is defined as one enzyme activity unit. When insoluble corncob xylan was employed as substrates, the recombinant xylanases were dissolved to 10 μM in the disodium hydrogen phosphate—citric acid buffer (pH 6.0, 200 mM). Then, 20 mg of insoluble corncob xylan was added to 1 mL of the xylanase solution and incubated at 37 °C for 6 h in a shaker at 200 rpm. Afterwards, 150μL of the supernatant was taken for the determination of enzyme activity using DNS reagent as mentioned above.

To investigate the optimal temperature for catalysis, the recombinant xylanases and glucuronoxylan were dissolved in the disodium hydrogen phosphate—citric acid buffer (pH 6.0, 200 mM), respectively. After that, 50 μL of recombinant xylanase solution (2 μM) was mixed with 100 μL of glucuronoxylan solution (0.2%) and incubated at gradient temperature (20–90 °C) for 20 min. Enzyme activities were then measured using DNS reagent as previously described. To investigate the optimal pH value for catalysis, the recombinant xylanases and glucuronoxylan were dissolved in the disodium hydrogen phosphate—citric acid buffer (pH 4.0–8.0, 200 mM) and the glycine—sodium hydroxide buffer (pH 8.0–11.0, 100 mM). After that, 50 μL of recombinant xylanase solution (2 μM) was mixed with 100 μL of glucuronoxylan solution (0.2%) and incubated at 50 °C for 20 min. The enzyme activities were then measured using the DNS reagent as described above.

To investigate the thermostability of the enzymes, the recombinant xylanases and glucuronoxylan were dissolved in the disodium hydrogen phosphate—citric acid buffer (pH 6.0, 200 mM), respectively. After that, 50 μL of recombinant xylanase solution (2 μM) was incubated at 40 °C, 50 °C, 60 °C or 70 °C for 1 h and then mixed with 100 μL of glucuronoxylan solution (0.2%), followed by the incubation at 50 °C for 20 min. The enzyme activities were then measured with DNS reagent as mentioned above.

To investigate the kinetic parameters, the recombinant xylanases and soluble xylans were dissolved in the disodium hydrogen phosphate—citric acid buffer (pH 6.0, 200 mM), respectively. After that, 50 μL of recombinant xylanase solution (2 μM) were mixed with 100 μL of xylan solution with gradient concentration (from 0.2 to 4%), followed by the incubation at 50 °C for 10 min. The enzyme activities were then measured using the DNS reagent as described above. The K_m_, V_max_ and k_cat_ values were calculated from the nonlinear regression curves using the OriginLab software package. All of these experiments were carried out in triplicate.

### The saccharification of lignocellulosic biomass

The saccharification of delignified corncob and Carolina poplar was carried out to study the stimulation of xylanases with or without CBMs to the hydrolysis of lignocellulosic biomass. In brief, 50 mg of delignified corncob or Carolina poplar powders with particle sizes under 75# mesh as well as 125 μL of commercial cellulase solution (2.5 mg/mL, C8546, Sigma-Aldrich) and of recombinant xylanase solution (25 μM) were loaded into a 5 mL tube. The reaction buffer (disodium hydrogen phosphate—citric acid buffer, pH 5.0, 200 mM) was then added to a final volume of 2.5 mL. Subsequently, the tubes were incubated in a shaker at 37 °C with a rotational speed of 200 rpm, and 10 μL of the supernatant were sampled at the 0th, 6th, 12th, 24th, 48th and 96th hour and then diluted to 150 μL for the determination of the reducing sugar concentration with DNS reagent. At the 96th hour, 100 μL of the supernatant was sampled and incubated in a boiling water bath for 20 min to stop the reaction. After filtration, the solution was loaded into a HPLC system (EClassic 3100, Elite) equipped with a MARS MOA 10u column and a refractive index detector to measure the glucose, xylose and arabinose concentration to calculate the cellulose and xylan conversion rates. The sulfuric acid solution (2.5 mM) was employed as mobile phase for separation at 60 °C with a flow rate of 0.6 mL/min.

### Phylogenetic analysis

The CBM sequences were collected from genbank database, and the CBM domains were predicted using dbCAN meta server and Conserved Domain Database tools [48, 49]. The CBM sequences were aligned and then used for the construction of phylogenetic tree with the neighbor-joining method using MEGA11 software package [50]. The p-distance and bootstrap method were employed for substitution model and phylogeny test, respectively. The sequences and other information of CBMs are accessible in Additional file 7 and Additional file 8.

### Structure analysis of CBMs

The 3D-structure of CrCBM13 was predicted using AlphaFold 2 [51]. The structure of Ara*f*-X3 was obtained from the crystal structure of SoCBM13-Ara*f*-X3 complex (PDB ID: 1V6V) [52]. After pretreatment, the Ara*f*-X3 was docked into the conserved binding site of CrCBM13 using AutoDock 4.2 [53]. The CBM structures were visualized using ChimeraX software package [54].

## Supplementary Information

**Additional file 1: Figure S1.** Quantitative analysis of binding ability by affinity gel electrophoresis.

**Additional file 2: **Predicted structures of CrCBM13.

**Additional file 3: Figure S2.** Sequence alignment of CBM2.

**Additional file 4: Table S1.** The kinetic parameters of rCrXyl30-FL and its CBM-truncated versions against glucuronoxylan.

**Additional file 5: Table S2.** The composition of delignified lignocellulosic biomass used in this study.

**Additional file 6: Table S3.** The primers used for gene cloning

**Additional file 7: Table S4.** The CBMs employed for constructing phylogenetic trees.

**Additional file 8: **Sequences of the CBMs employed for constructing phylogenetic trees.

## Data Availability

All data generated during this study are included in the article and additional files. Raw data used and/or analysed during the current study are available from the corresponding author on reasonable request.

## Abbreviations

Ara*f*-X3: 3(2)-Alpha-l-arabinofuranosyl-xylotriose
BSA: Bovine albumin
CBM: Carbohydrate-binding module
CD: Catalytic domain
CEL: Commercial cellulase
DNS: Dinitrosalicylic acid
FL: Full-length
GH: Glycoside hydrolase
K_d_: Dissociation constant
(Me)GA: d-glucuronic acid or 4-o-methyl-d-glucuronic acid
PAGE: Polyacrylamide gel electrophoresis
