# Supplementary figures and images for: Functional identification of two novel carbohydrate-binding modules of glucuronoxylanase CrXyl30 and their contribution to the lignocellulose saccharification
Source: Biotechnol Biofuels Bioprod. 2023 Mar 8;16:40. doi: 10.1186/s13068-023-02290-7 (PMC9996879; doi:10.1186/s13068-023-02290-7)

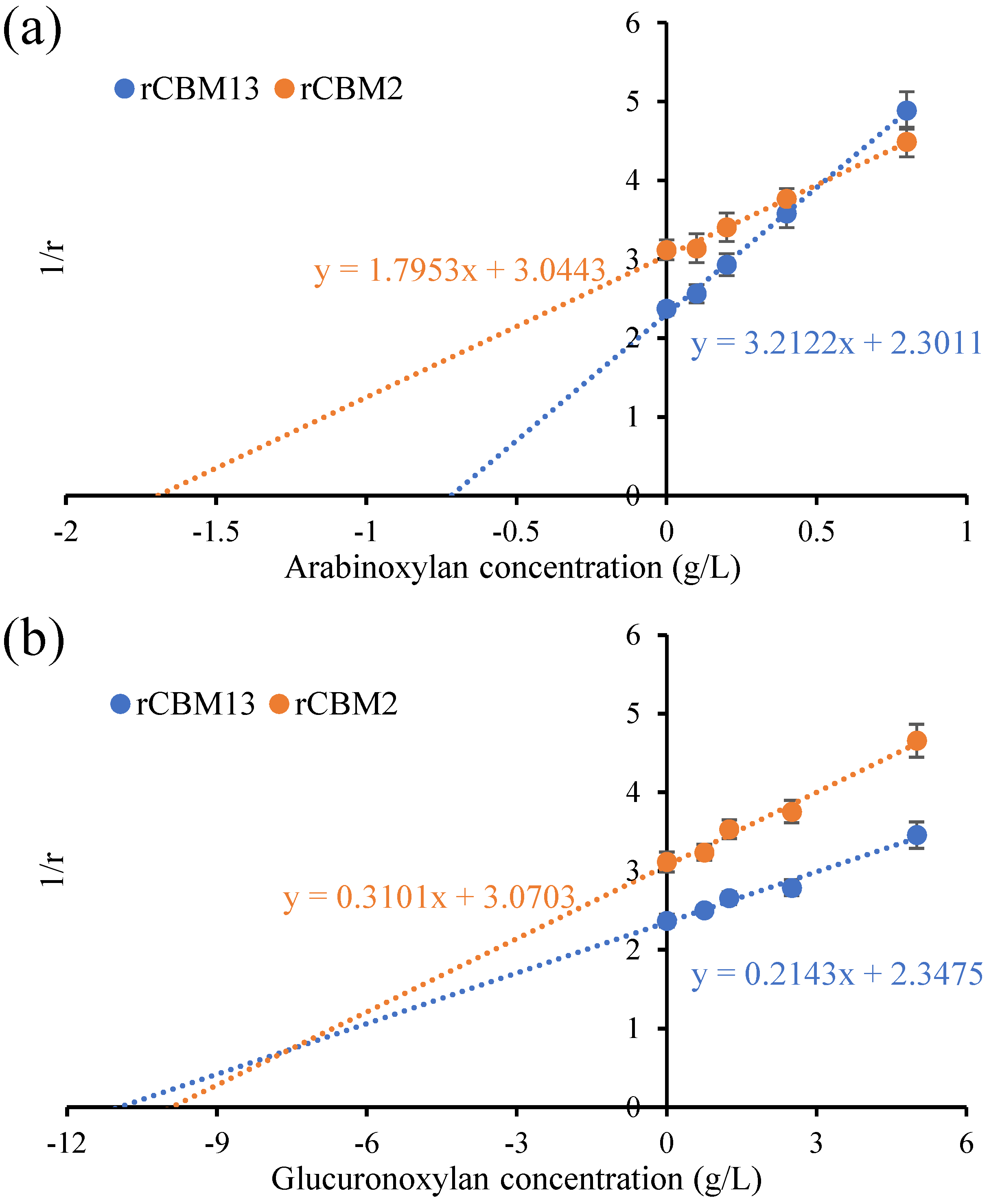

Supplement: Supplementary file 1 — Additional file 1: Figure S1. Quantitative analysis of binding ability by affinity gel electrophoresis. [file 13068_2023_2290_MOESM1_ESM.tiff]

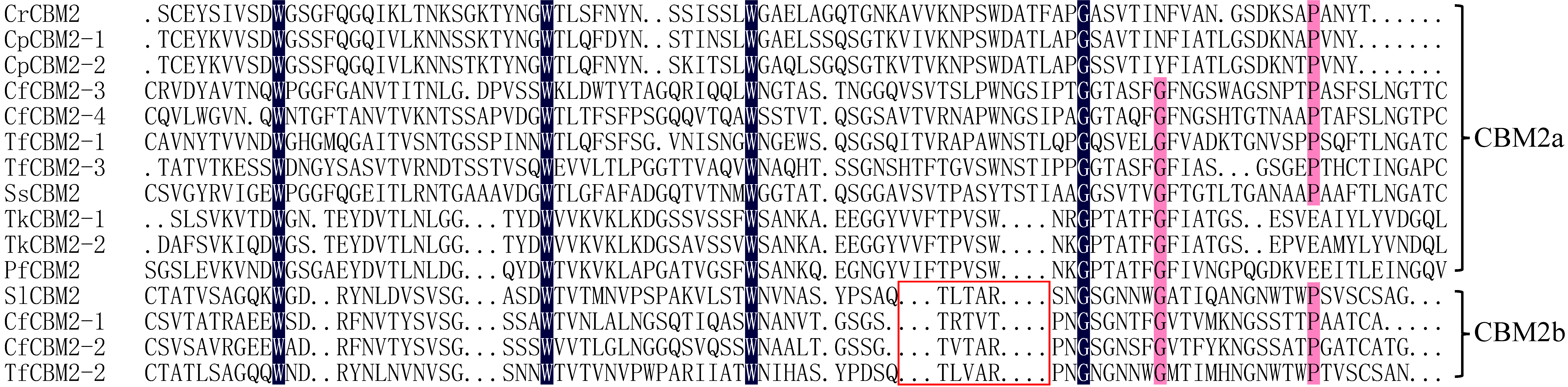

Supplement: Supplementary file 3 — Additional file 3: Figure S2. Sequence alignment of CBM2. [file 13068_2023_2290_MOESM3_ESM.tiff]
