# Supplementary material for: Functional identification of two novel carbohydrate-binding modules of glucuronoxylanase CrXyl30 and their contribution to the lignocellulose saccharification
Source: Biotechnol Biofuels Bioprod. 2023 Mar 8;16:40. doi: 10.1186/s13068-023-02290-7 (PMC9996879; doi:10.1186/s13068-023-02290-7)
Supplement: Supplementary file 4 — Additional file 4: Table S1. The kinetic parameters of rCrXyl30-FL and its CBM-truncated versions against glucuronoxylan. [file 13068_2023_2290_MOESM4_ESM.docx]

**Table S1 The kinetic parameters of rCrXyl30-FL and its CBM-truncated versions against glucuronoxylan**

|  | rCrXyl30-FL | rCrXyl30-CBM13 | rCrXyl30-CD |
| --- | --- | --- | --- |
| K_m_ (g/L) | 0.7 ± 0.0^a^ | 2.0 ± 0.1^b^ | 1.6 ± 0.1^c^ |
| k_cat_ (/s) | 11.8 ± 0.4^A^ | 5.5 ± 0.2^B^ | 6.4 ± 0.2^C^ |
| k_cat_/K_m_ (L/g/s) | 16.3 ± 1.3^α^ | 2.8 ± 0.2^β^ | 4.1 ± 0.3^γ^ |

Different superscripts indicate significant differences (*P* < 0.05).
