# Supplementary material for: Functional identification of two novel carbohydrate-binding modules of glucuronoxylanase CrXyl30 and their contribution to the lignocellulose saccharification
Source: Biotechnol Biofuels Bioprod. 2023 Mar 8;16:40. doi: 10.1186/s13068-023-02290-7 (PMC9996879; doi:10.1186/s13068-023-02290-7)
Supplement: Supplementary file 5 — Additional file 5: Table S2. The composition of delignified lignocellulosic biomass used in this study. [file 13068_2023_2290_MOESM5_ESM.docx]

**Table S2 The composition of delignified lignocellulosic biomass used in this study**

|  | Cellulose | Hemicellulose | Lignin |
| --- | --- | --- | --- |
| Corncob | 37.72 ± 2.74 | 48.20 ± 3.92 | 0.72 ± 0.22 |
| Carolina poplar | 60.62 ± 3.55 | 24.61 ± 1.77 | 1.21 ± 0.39 |

Unit: %
