# Supplementary material for: Functional identification of two novel carbohydrate-binding modules of glucuronoxylanase CrXyl30 and their contribution to the lignocellulose saccharification
Source: Biotechnol Biofuels Bioprod. 2023 Mar 8;16:40. doi: 10.1186/s13068-023-02290-7 (PMC9996879; doi:10.1186/s13068-023-02290-7)
Supplement: Supplementary file 6 — Additional file 6: Table S3. The primers used for gene cloning [file 13068_2023_2290_MOESM6_ESM.docx]

**Table S3 The primers used for gene cloning**

| **Gene** | **Endonucleases** | **Sequence** |
| --- | --- | --- |
| rCrXyl30-FL | *Nhe* I | CTAGCTAGCGCCAGCGATGTAACCGTCAATT |
|  | *Xho* I | CCGCTCGAGATATTTTAGCCAAATGTATAAT |
| rCrXyl30-CBM13 | *Nhe* I | CTAGCTAGCGCCAGCGATGTAACCGTCAATT |
|  | *Xho* I | CCGCTCGAGTTAACTTACGATGGAATACTCACAGGA |
| rCrXyl30-CD | *Nhe* I | CTAGCTAGCGCCAGCGATGTAACCGTCAATT |
|  | *Xho* I | CCGCTCGAGTCAGTATAAACCATCTTCTAATTT |
| rCBM13-CBM2 | *Nhe* I | CTAGCTAGCACACCACAGCCTTCTACACAGCCA |
|  | *Xho* I | CCGCTCGAGATATTTTAGCCAAATGTATAAT |
| rCBM13 | *Nhe* I | CTAGCTAGCACACCACAGCCTTCTACACAGCCA |
|  | *Xho* I | CCGCTCGAGTTAACTTACGATGGAATACTCACAGGA |
| rCBM2 | *Nhe* I | CTAGCTAGCCCAAGTCAGGAACCAAGTCAGA |
|  | *Xho* I | CCGCTCGAGATATTTTAGCCAAATGTATAAT |
| rSaXyl10 | *Bam*H I | ACAGCTGGATCCAAGGAAGGACCGGAAGCA |
|  | *Hin*d III | TCAAGCAAGCTTTTAATCAATAATTCTCCAGTAAGCAG |
| rSaXyl10-CBM13-CBM2 | *Bam*H I | ACAGCTGGATCCAAGGAAGGACCGGAAGCA |
|  |  | ACAGTATTTCCTGAAATAACATCAATAATTCTCCAGTA |
|  |  | TACTGGAGAATTATTGATGTTATTTCAGGAAATACTGT |
|  | *Xho* I | CCGCTCGAGATATTTTAGCCAAATGTATAAT |
| rSaXyl10-CBM13 | *Bam*H I | ACAGCTGGATCCAAGGAAGGACCGGAAGCA |
|  |  | ACAGTATTTCCTGAAATAACATCAATAATTCTCCAGTA |
|  |  | TACTGGAGAATTATTGATGTTATTTCAGGAAATACTGT |
|  | *Xho* I | CCGCTCGAGTTAACTTACGATGGAATACTCACAGGA |
| rSaXyl10-CBM2 | *Bam*H I | ACAGCTGGATCCAAGGAAGGACCGGAAGCA |
|  |  | GAGATTCCGCTTGGAAGATCAATAATTCTCCAGTA |
|  |  | TACTGGAGAATTATTGATCTTCCAAGCGGAATCT |
|  | *Xho* I | CCGCTCGAGATATTTTAGCCAAATGTATAAT |
