# Supplementary material for: Functional identification of two novel carbohydrate-binding modules of glucuronoxylanase CrXyl30 and their contribution to the lignocellulose saccharification
Source: Biotechnol Biofuels Bioprod. 2023 Mar 8;16:40. doi: 10.1186/s13068-023-02290-7 (PMC9996879; doi:10.1186/s13068-023-02290-7)
Supplement: Supplementary file 7 — Additional file 7: Table S4. The CBMs employed for constructing phylogenetic trees. [file 13068_2023_2290_MOESM7_ESM.docx]

**Table S4 The CBMs employed for constructing phylogenetic trees**

| **Family/Name** | **Accession number** | **Ligand^a^** | **Enzyme type** | **Microbial source** | **References** |
| --- | --- | --- | --- | --- | --- |
| CrCBM2 | OP172628 | Cellulose | Xylanase | *Cellulosilyticum ruminicola* | This study |
| CfCBM2-1 | AEE44519.1 | Xylan | Xylanase | *Cellulomonas fimi* | [1, 2] |
| CfCBM2-2 | AEE44519.1 | Xylan | Xylanase | *Cellulomonas fimi* | [1, 2] |
| CfCBM2-3 | AEE47298.1 | Cellulose | Cellulase | *Cellulomonas fimi* | [3] |
| CfCBM2-4 | AEA30147.1 | Cellulose | Xylanase | *Cellulomonas fimi* | [4] |
| CpCBM2-1 | SFS02053.1 | Unidentified | Xylanase | *Clostridium populeti* |  |
| CpCBM2-2 | WP_092562273.1 | Unidentified | Cellulase | *Clostridium populeti* |  |
| TfCBM2-1 | QOS59399.1 | Cellulose | Xylanase | *Thermobifida fusca* | [5] |
| TfCBM2-2 | QOS57860.1 | Cellulose | Xylanase | *Thermobifida fusca* | [6] |
| TfCBM2-3 | QOS57567.1 | Unidentified | Cellulase | *Thermobifida fusca* |  |
| TkCBM2-1 | BAD85954.1 | Cellulose/Chitin | Chitinase | *Thermococcus kodakarensis* | [7] |
| TkCBM2-2 | BAD85954.1 | Cellulose/Chitin | Chitinase | *Thermococcus kodakarensis* | [7] |
| PfCBM2 | QEK78877.1 | Chitin/Xyloglucan | Chitinase | *Pyrococcus furiosus* | [8, 9] |
| SlCBM2 | AIJ16163.1 | Unidentified | Xylanase | *Streptomyces lividans* |  |
| SsCBM2 | WP_014046222.1 | Cellulose/Galactomannan | Mannanase | *Streptomyces* sp. SirexAA-E | [10] |
| PbCBM35 | 4QB1_A | Xylan | Xylanase | *Paenibacillus barcinonensis* | [11] |
| CrCBM13 | OP172628 | Xylan | Xylanase | *Cellulosilyticum ruminicola* | This study |
| CcCBM13-1 | QUB98532.1 | Unidentified | Glycoside hydrolase family 30 | *Cellulosimicrobium cellulans* |  |
| CcCBM13-2 | QUB99327.1 | Laminarin | β-1,3-glucanase | *Cellulosimicrobium cellulans* | [12] |
| CtCBM13-1 | ABN53395.1 | Unidentified | Arabinoxylanase | *Clostridium thermocellum* |  |
| CtCBM13-2 | ADU74624.1 | Galactose/Galactan | exo-β-1,3-galactanase | *Clostridium thermocellum* | [13] |
| CpCBM13 | SFS02053.1 | Unidentified | Xylanase | *Clostridium populeti* |  |
| CsCBM13 | KSW17752.1 | Unidentified | α-L-arabinofuranosidase | *Cellulomonas* sp. B6 |  |
| LpCBM13 | ABX43935.1 | Unidentified | Arabinogalactan endo-1,4-β-galactosidase | *Lachnoclostridium phytofermentans* |  |
| LtLectin^b^ | BAA36393.1 | Galactose/Lactose | Nonenzyme | *Lumbricus terrestris* | [14] |
| PhCBM13 | AAF86343.2 | Unidentified | Pectate lyase | *Pseudoalteromonas haloplanktis* |  |
| SaCBM13 | BAC69897.1 | Arabinose | β-L-arabinopyranosidase | *Streptomyces avermitilis* | [15] |
| SlCBM13 | AAC26525.1 | Xylan | Xylanase | *Streptomyces lividans* | [16] |
| SoCBM13 | 1ISV_A | Xylan | Xylanase | *Streptomyces olivaceoviridis* | [17] |
| SsCBM13 | ACF57946.1 | Insoluble xylan/  Microcrystalline cellulose | Xylanase | *Streptomyces* sp. S27 | [18] |
| AkCBM42 | 1WD3_A | Arabinose | α-L-arabinofuranosidase | *Aspergillus kawachii* | [19] |

^a^ Ligands of these CBMs were indicated by references.

^b^ The R-type lectin from *Lumbricus terrestris* belongs to CBM13.

Reference

1. Simpson PJ, Bolam DN, Cooper A, Ciruela A, Hazlewood GP, Gilbert HJ, Williamson MP. **A family IIb xylan-binding domain has a similar secondary structure to a homologous family IIa cellulose-binding domain but different ligand specificity.** *Structure.* 1999;**7:**853-864.

2. Bolam DN, Xie HF, White P, Simpson PJ, Hancock SM, Williamson MP, Gilbert HJ. **Evidence for synergy between family 2b carbohydrate binding modules in *Cellulomonas fimi* xylanase 11A.** *Biochemistry.* 2001;**40:**2468-2477.

3. Simpson PJ, Xie HF, Bolam DN, Gilbert HJ, Williamson MP. **The structural basis for the ligand specificity of family 2 carbohydrate-binding modules.** *J Biol Chem.* 2000;**275:**41137-41142.

4. Xu GY, Ong E, Gilkes NR, Kilburn DG, Muhandiram DR, Harrisbrandts M, Carver JP, Kay LE, Harvey TS. **Solution structure of a cellulose-binding domain from *Cellulomonas fimi* by nuclear-magnetic-resonance spectroscopy.** *Biochemistry.* 1995;**34:**6993-7009.

5. Wu XY, Shi ZL, Tian WY, Liu MY, Huang SX, Liu XL, Yin H, Wang LS. **A thermostable and CBM2-linked GH10 xylanase from *Thermobifida fusca* for paper bleaching.** *Front Bioeng Biotechnol.* 2022;**10**:939550.

6. Irwin D, Jung ED, Wilson DB. **Characterization and sequence of a *Thermomonospora fusca* xylanase.** *Appl Environ Microbiol.* 1994;**60:**763-770.

7. Hanazono Y, Takeda K, Niwa S, Hibi M, Takahashi N, Kanai T, Atomi H, Miki K. **Crystal structures of chitin binding domains of chitinase from *Thermococcus kodakarensis* KOD1.** *FEBS Lett.* 2016;**590:**298-304.

8. Nakamura T, Mine S, Hagihara Y, Ishikawa K, Ikegami T, Uegaki K. **Tertiary structure and carbohydrate recognition by the chitin-binding domain of a hyperthermophilic chitinase from *Pyrococcus furiosus*.** *J Mol Biol.* 2008;**381:**670-680.

9. Hernandez-Gomez MC, Rydahl MG, Rogowski A, Morland C, Cartmell A, Crouch L, Labourel A, Fontes CMGA, Willats WGT, Gilbert HJ, Knox JP. **Recognition of xyloglucan by the crystalline cellulose-binding site of a family 3a carbohydrate-binding module.** *FEBS Lett.* 2015;**589:**2297-2303.

10. Takasuka TE, Acheson JF, Bianchetti CM, Prom BM, Bergeman LF, Book AJ, Currie CR, Fox BG. **Biochemical properties and atomic resolution structure of a proteolytically processed beta-mannanase from cellulolytic *Streptomyces* sp. SirexAA-E.** *PLoS One.* 2014;**9**: e94166.

11. Sainz-Polo MA, Valenzuela SV, Gonzalez B, Pastor FIJ, Sanz-Aparicio J. **Structural analysis of glucuronoxylan-specific Xyn30D and its attached CBM35 domain gives insights into the role of modularity in specificity.** *J Biol Chem.* 2014;**289:**31088-31101.

12. Tamashiro T, Tanabe Y, Ikura T, Ito N, Oda M. **Critical roles of Asp270 and Trp273 in the alpha-repeat of the carbohydrate-binding module of endo-1,3-beta-glucanase for laminarin-binding avidity.** *Glycoconjugate J.* 2012;**29:**77-85.

13. Jiang DH, Fan JP, Wang XP, Zhao Y, Huang B, Liu JF, Zhang XJC. **Crystal structure of 1,3Gal43A, an exo-beta-1,3-galactanase from *Clostridium thermocellum*.** *J Struct Biol.* 2012;**180:**447-457.

14. Hemmi H, Kuno A, Hirabayashi J. **NMR structure and dynamics of the C-terminal domain of R-type lectin from the earthworm *Lumbricus terrestris*.** *FEBS J.* 2013;**280:**70-82.

15. Ichinose H, Fujimoto Z, Honda M, Harazono K, Nishimoto Y, Uzura A, Kaneko S. **A beta-L-arabinopyranosidase from *Streptomyces avermitilis* is a novel member of glycoside hydrolase family 27.** *J Biol Chem.* 2009;**284:**25097-25106.

16. Boraston AB, Tomme P, Amandoron EA, Kilburn DG. **A novel mechanism of xylan binding by a lectin-like module from *Streptomyces lividans* xylanase 10A.** *Biochem J.* 2000;**350 Pt 3:**933-941.

17. Fujimoto Z, Kaneko S, Kuno A, Kobayashi H, Kusakabe I, Mizuno H. **Crystal structures of decorated xylooligosaccharides bound to a family 10 xylanase from *Streptomyces olivaceoviridis* E-86.** *J Biol Chem.* 2004;**279:**9606-9614.

18. Li N, Shi PJ, Yang PL, Wang YR, Luo HY, Bai YG, Zhou ZG, Yao B. **A xylanase with high pH stability from *Streptomyces* sp. S27 and its carbohydrate-binding module with/without linker-region-truncated versions.** *Appl Microbiol Biotechnol.* 2009;**83:**99-107.

19. Miyanaga A, Koseki T, Matsuzawa H, Wakagi T, Shoun H, Fushinobu S. **Crystal structure of a family 54 alpha-L-arabinofuranosidase reveals a novel carbohydrate-binding module that can bind arabinose.** *J Biol Chem.* 2004;**279:**44907-44914.
