# Supplementary material for: Functional identification of two novel carbohydrate-binding modules of glucuronoxylanase CrXyl30 and their contribution to the lignocellulose saccharification
Source: Biotechnol Biofuels Bioprod. 2023 Mar 8;16:40. doi: 10.1186/s13068-023-02290-7 (PMC9996879; doi:10.1186/s13068-023-02290-7)
Supplement: Supplementary file 8 — Additional file 8: Sequences of the CBMs employed for constructing phylogenetic trees. [file 13068_2023_2290_MOESM8_ESM.docx]

>CrCBM2

SCEYSIVSDWGSGFQGQIKLTNKSGKTYNGWTLSFNYNSSISSLWGAELAGQTGNKAVVKNPSWDATFAPGASVTINFVANGSDKSAPANYT

>CfCBM2-1

CSVTATRAEEWSDRFNVTYSVSGSSAWTVNLALNGSQTIQASWNANVTGSGSTRTVTPNGSGNTFGVTVMKNGSSTTPAATCA

>CfCBM2-2

CSVSAVRGEEWADRFNVTYSVSGSSSWVVTLGLNGGQSVQSSWNAALTGSSGTVTARPNGSGNSFGVTFYKNGSSATPGATCATG

>CfCBM2-3

CRVDYAVTNQWPGGFGANVTITNLGDPVSSWKLDWTYTAGQRIQQLWNGTASTNGGQVSVTSLPWNGSIPTGGTASFGFNGSWAGSNPTPASFSLNGTTC

>CfCBM2-4

CQVLWGVNQWNTGFTANVTVKNTSSAPVDGWTLTFSFPSGQQVTQAWSSTVTQSGSAVTVRNAPWNGSIPAGGTAQFGFNGSHTGTNAAPTAFSLNGTPC

>TfCBM2-1

CAVNYTVVNDWGHGMQGAITVSNTGSSPINNWTLQFSFSGVNISNGWNGEWSQSGSQITVRAPAWNSTLQPGQSVELGFVADKTGNVSPPSQFTLNGATC

>TfCBM2-2

CTATLSAGQQWNDRYNLNVNVSGSNNWTVTVNVPWPARIIATWNIHASYPDSQTLVARPNGNGNNWGMTIMHNGNWTWPTVSCSAN

>TfCBM2-3

TATVTKESSWDNGYSASVTVRNDTSSTVSQWEVVLTLPGGTTVAQVWNAQHTSSGNSHTFTGVSWNSTIPPGGTASFGFIASGSGEPTHCTINGAPC

>CpCBM2-1

TCEYKVVSDWGSSFQGQIVLKNNSSKTYNGWTLQFDYNSTINSLWGAELSSQSGTKVIVKNPSWDATLAPGSAVTINFIATLGSDKNAPVNY

>CpCBM2-2

TCEYKVVSDWGSSFQGQIVLKNNSTKTYNGWTLQFNYNSKITSLWGAQLSGQSGTKVTVKNPSWDATLAPGSSVTIYFIATLGSDKNTPVNY

>TkCBM2-1

SLSVKVTDWGNTEYDVTLNLGGTYDWVVKVKLKDGSSVSSFWSANKAEEGGYVVFTPVSWNRGPTATFGFIATGSESVEAIYLYVDGQLW

>TkCBM2-2

DAFSVKIQDWGSTEYDVTLNLGGTYDWVVKVKLKDGSAVSSVWSANKAEEGGYVVFTPVSWNKGPTATFGFIATGSEPVEAMYLYVNDQLW

>SlCBM2

CTATVSAGQKWGDRYNLDVSVSGASDWTVTMNVPSPAKVLSTWNVNASYPSAQTLTARSNGSGNNWGATIQANGNWTWPSVSCSAG

>SsCBM2

CSVGYRVIGEWPGGFQGEITLRNTGAAAVDGWTLGFAFADGQTVTNMWGGTATQSGGAVSVTPASYTSTIAAGGSVTVGFTGTLTGANAAPAAFTLNGATC

>PfCBM2

SGSLEVKVNDWGSGAEYDVTLNLDGQYDWTVKVKLAPGATVGSFWSANKQEGNGYVIFTPVSWNKGPTATFGFIVNGPQGDKVEEITLEINGQVI

>PbCBM35

YEAETGTTLTDAVVETLYPGYTGSGYVNFNAYTNSAIEWNAINNMTTGTKNVKFRYALESGTRNLDIYVNGTKVLSNEPFTETGSWSTWGEKTIQVAMNSGVNTLRIVTTGTEGPNMDNITV

>CrCBM13

LYYIKNVNAQKYLQVKDNTGKNVQNVELGAGSGAAGQKWYLSNTSDGYITLKSSLGDFMLDIGNGVDEDGANVQIYASHGGAAQQFQVKSASGSNGYIIATKASSTTKALDASENGTADGTNVIQWTINGKANQQWIFEKVNETSVSPQ

>SoCBM13

GQIKGVGSGRCLDVPNASTTDGTQVQLYDCHSATNQQWTYTDAGELRVYGDKCLDAAGTGNGTKVQIYSCWGGDNQKWRLNSDGSIVGVQSGLCLDAVGGGTANGTLIQLYSCSNGSNQRWT

>SlCBM13

QIKGVGSGRCLDVPDASTSDGTQLQLWDCHSGTNQQWAATDAGELRVYGDKCLDAAGTSNGSKVQIYSCWGGDNQKWRLNSDGSVVGVQSGLCLDAVGNGTANGTLIQLYTCSNGSNQRWT

>CsCBM13

ALDVYNLAMNDGARITQWTRNDGQQQQWQFVDSGNGDYRIKSRLSGKVLDVYNWSTADGAAINQYTDRDQANQRFRLQDAGDGYVILVNRQSNKAVEVQGGSTADGANVVQYSNWGGANQ

>SsCBM13

TIKGVGSGRCLDVPNASTTDGTQLHLWDCHNGTNQQWTYTNAGELRVYGNKCLDAAGTGNGAKVQIYSCWGGDNQKWRLNSDGSIVGVQSGLCLDAVGAGTANGTLIQLYSCSNGSNQRWT

>CpCBM13

DGWYYIKNVNAQKYLQVKDNTGAAGQNVEISTGTGAAGQKWYLTNTSDGYITLTSGLGNYMMDVANASDTDGANVQIYNGYSGNAQKFVIKSTSTSNVYTVATKASNGTKMLDAYNFGKTDGTNVCQWTYGGYANQQWVFESTSS

>CtCBM13-1

TGTIPDGTYKFLNRANGKTLQEVTGNNSIITADYKGITEQHWKIQHIGGGQYRISSAGRGWNWNWWMGFGTVGWWGTGSSTCFIISPTGDGYYRIVLVGDGTNLQISSGDPSKIEGKAFHGGANQQWAILPVSA

>LtLectin

PKFFYIKSELNGKVLDIEGQNPAPGSKIITWDQKKGPTAVNQLWYTDQQGVIRSKLNDFAIDASHEQIETQPFDPNNPKRAWIVSGNTIAQLSDRDIVLDIIKSDKEAGAHICAWKQHGGPNQKFIIE

>CcCBM13-1

GTGVVIRTDAPVAEQAWELTALGAPEGSGTHRTRYAVTNAATGRQLAVAADTSAVLQDAPADVADTPLAAQWILSTTGDGTFTLVNASSKTLLEVGGQATADGSPVGTYLANSGVNQRWRVVDETVLGTEPVQAFTTPGTAPELPATVT

>CtCBM13-2

TRYKLVNKNSGKVLDVLDGSVDNAAQIVQWTDNGSLSQQWYLVDVGGGYKKIVNVKSGRALDVKDESKEDGGVLIQYTSNGGYNQHWKFTDIGDGYYKISSRHCGKLIDVRKWSTEDGGIIQQWSDAGGTNQHWKLV

>SaCBM13

DLTTMTSETAGILKNPEVIAVDQDSRGLQGVKVAEDTTGLQAYGKVLSGTGNRAVVLLNRTSAAHDITVRWSDLGLTNASATVRDLWARQNVGTSATGYTASVPAGGSVMLTVTGGTEAAGGAYAATSTGRYTGVTAA

>CcCBM13-2

VRAANGMCVDVPWADPTDGNQVQIVTCSGNAAQTWTRGSDGTVRALGKCLDVRDGATTRGAAVQVWTCNGTGAQKWAYDAGSKALRNPQSGLCLDAAGGAPLHDGQRLQTWTCNGTTAQQWT

>PhCBM13

VNGVYRVTPKHSAKSLDVANCANSNGANISQWSWLNNDCQKFNISTVDGIWHRISPVNAPSKGLDVAANSTVNGANIALYTYTGSYNQQFRFQAAGTGKWRIINRNSELCFDIEGNKANDGANLLQWTCSAGSENQMFELT

>LpCBM13

RGGSTSDNAVLEQYSYGAWSSQKWTFSLNSSGYYTIKSVLSSKVMDVSGASTSEGASVIQYTSNNGNNQQWSLGSTGDGYYKLINRGSGKLLAVQNASTEEGVALVQQTDTNALSQMWRL

>AkCBM42

SLRVTTPGYTTRYIAHTDTTVNTQVVDDDSSTTLKEEASWTVVTGLANSQCFSFESVDTPGSYIRHYNFELLLNANDGTKQFHEDATFCPQAALNGEGTSLRSWSYPTRYFRHYENVLYAASNGGVQTFDSKTSFNNDVSFE
